# Impact of Cyberchondria on Health and Quality of Life: Scoping Review

**DOI:** 10.2196/77977

**Published:** 2025-12-04

**Authors:** Chenxi Yang, Richard Huan Xu

**Affiliations:** 1 Department of Rehabilitation Sciences Faculty of Health and Social Sciences Hong Kong Polytechnic University Kowloon China (Hong Kong)

**Keywords:** cyberchondria, quality of life, physical health, mental health, social well-being, cognition, health-related behavior

## Abstract

**Background:**

Cyberchondria is often associated with psychological distress, straining doctor-patient relationships, and financial burdens. Over the past few decades, increasing research has explored its associations with quality of life (QoL). However, existing reviews have not comprehensively synthesized or narratively analyzed these connections.

**Objective:**

This study aims to consolidate current research, identify key trends, and examine how cyberchondria affects QoL, while providing insights for future research directions.

**Methods:**

The literature search was conducted on 4 databases PsycINFO, PubMed, CINAHL, and Web of Science. The review was restricted to peer-reviewed journals published in English from inception to October 9, 2025. The inclusion criteria were as follows (1) original studies examining health-related factors associated with cyberchondria, (2) participants of any demographic, and (3) English-language full texts. Studies were excluded if they assessed health anxiety as a representation of cyberchondria. The Newcastle-Ottawa Scale for cross-sectional studies was used to assess the risk of bias in the included studies. Narrative analysis was used for data synthesis. This review was reported in accordance with the PRISMA-ScR (Preferred Reporting Items for Systematic Reviews and Meta-Analyses extension for Scoping Reviews) checklist.

**Results:**

A total of 9483 records were identified from 4 databases, with 87 studies meeting the inclusion criteria for this review. All of the included studies used a cross-sectional design. Most of the included studies were rated as moderate risk (54.4%) to low risk (36.7%). Correlations were found between cyberchondria and QoL domains, including physical health (eg, pain and discomfort, sleep quality), psychological health (eg, anxiety, fear, negative feelings or emotions, anxiety sensitivity, intolerance of uncertainty, obsessive-compulsive symptoms, and depression), level of independence (eg, usual or daily activities, and mobility), social relationship (eg, personal relationship, communication, and social support), environment (eg, eHealth literacy and financial satisfaction), and behavior (eg, addictive behavior).

**Conclusions:**

This scoping review synthesizes key risk factors and challenges influencing the QoL in individuals with cyberchondria. The findings emphasize the need for clinicians to adopt a holistic approach to assess and manage cyberchondria, addressing its multifaceted impact on QoL.

## Introduction

Cyberchondria refers to excessive or repeated online health information seeking (OHIS) that persists despite negative consequences and is associated with increased health anxiety [1]. Cyberchondria is considered an abnormal behavioral pattern rather than a condition or diagnostic entity, and is especially common among people with high levels of health anxiety [2]. The rise of cyberchondria is closely linked to the digital revolution, online health information management issues (ie, difficulty in managing the abundance of online health information or information overload, especially when that information is ambiguous, inconsistent, or conflicting), and maladaptive interactions with the internet (eg, unrealistic expectations of the internet and erroneous beliefs about the ranking of OHIS results) [3].

With increasing internet access and reliance on digital health resources, cyberchondria has emerged as a significant global public health concern [1]. It not only affects the individuals’ health [3,4], but also impacts the delivery of health services [5,6]. Additionally, individuals with cyberchondria tend to overrely on online information for self-diagnosis, often resulting in tense doctor-patient relationships through disagreements with the judgment or decision of health care professionals [7,8]. Furthermore, individuals who worry excessively about their health may misinterpret benign symptoms as serious illnesses [9,10]. This can lead them to seek unnecessary medical tests or treatments, thus straining health care systems and increasing social costs [11].

Recently, the number of studies on the impact of cyberchondria on health has increased significantly. Two meta-analyses confirmed a positive correlation between health anxiety and cyberchondria [3,12]. McMullan et al [3] identified age as a moderator of this association’s strength. Meanwhile, Schenkel et al [12], systematically reviewed evidence regarding the association between cyberchondria and intolerance of uncertainty, obsessive-compulsive symptoms, and anxiety sensitivity. These findings provide evidence that cyberchondria has a strong emotional basis. Recent reviews have explored the potential classification of cyberchondria, examining whether it is a behavioral addiction [13] or a subtype of obsessive-compulsive disorder [14]. Additionally, Vismara et al [15] provided an overview of updates in cyberchondria research, specifically focusing on studies conducted during the COVID-19 pandemic. Furthermore, 2 reviews offered a comprehensive analysis of cyberchondria, including its theoretical foundations, assessment tools, prevention and management strategies, and relationships with other constructs [1,16].

Quality of life (QoL) is increasingly identified as a crucial indicator of health and well-being that contributes to an individual’s overall satisfaction and function in life [17]. In health care, assessing QoL provides valuable insights into the effectiveness of treatments and supports holistic approaches to patient care [18]. Emerging evidence suggests that cyberchondria can significantly affect QoL [19]; yet, there is a lack of comprehensive reviews and analyses that consolidate the findings on how cyberchondria specifically impacts QoL. As QoL is now recognized as a critical outcome in clinical decision-making [20], a focused synthesis is needed to clarify how cyberchondria impacts QoL and related health factors. Such a review can inform the development of evidence-based guidelines and interventions, especially as digital health resources become increasingly accessible [21]. Therefore, this review has two objectives: (1) summarize key discoveries and highlight emerging trends in the study of cyberchondria by addressing the central question, “What health-related factors are associated with cyberchondria, and how do they influence one another?” and (2) offer insights into the complex interplay between cyberchondria and QoL while identifying areas for future research.

## Methods

### Study Design

The PRISMA-ScR (Preferred Reporting Items for Systematic Reviews and Meta-Analyses extension for Scoping Reviews) checklist [22] was followed for this study (Multimedia Appendix 1).

### Eligibility Criteria

Eligible studies were required to meet the following criteria: (1) address health-related factors that potentially interact with cyberchondria; (2) involve participants of any age, sex, ethnicity, or clinical background; (3) be published in English; and (4) have accessible full text. Conference abstracts, research protocols, dissertations, editorials, and review articles were excluded. Studies that solely used health anxiety to quantify cyberchondria were also excluded to maintain the conceptual integrity of the term. The eligibility criteria following the PICO framework (Participants, Interventions, Comparisons, and Outcomes) [23] are presented in Table 1.

**Table 1:** Eligibility criteria for retrieved studies.

Elements	Inclusion criteria	Exclusion criteria
Population	People of any age, sex, ethnicity, or clinical background	—^a^
Intervention or exposure	None (no restrictions applied)	—
Comparator	None (no restrictions applied)	—
Outcome	Cyberchondria and any health-related outcomes, including health status, health-related quality of life, and health-risk factors ( physical activity, diet, smoking, alcohol consumption, etc)	Only use measurements evaluated health anxiety to quantify cyberchondria
Study design	Quantitative (eg, RCTs^b^, quasiexperimental, cross-sectional, longitudinal, cohort, and case-control) or qualitative (eg, interviews, focus groups, and case reports)	Reviews, systematic reviews, editorials, commentaries, letters, conference abstracts, protocols.
Language	Published in English	—

^a^Not applicable.

^b^RCT: randomized controlled trial.

### Search Strategy

Two reviewers (CY and RX) independently conducted a comprehensive literature search using the following four electronic databases: PsycINFO, PubMed, CINAHL, and Web of Science. The search covered publications from inception to October 9, 2025, and included three groups of search terms (1) cyberchondria: cyberchondria*, (2) cyber (eg, internet, online, and web-based), and (3) hypochondria (eg, hypochondria*, health anxiety, and illness anxiety). The reference lists of relevant studies were manually searched. The search strategy is detailed in Multimedia Appendix 2.

### Study Selection

The entire process of the study selection was conducted using EndNote 21 (Clarivate) [24]. Study selection was independently conducted by 2 reviewers (CY and RX), and any discrepancies were resolved through reviewing the full text and discussion among reviewers. The Cohen κ was used to evaluate the interrater reliability of the study selection result [25]. The article selection process consisted of 3 phases the removal of duplicates, screening of titles and abstracts, and full-text screening. Following the automatic removal of duplicates using EndNote 21, we conducted title and abstract screening to exclude review articles and protocols while assessing whether the studies explored the role of cyberchondria in health outcomes. Subsequently, the remaining studies underwent full-text screening to confirm compliance with all eligibility criteria.

### Risk of Bias

The risk of bias in the included studies was assessed using the Newcastle-Ottawa Scale for cross-sectional studies (NOS-xs) [26]. This scale consists of 3 domains study sample selection (maximum of 2 points), assessment of exposure and outcome (maximum of 4 points), and confounding factors (maximum of 3 points), with each domain containing 2 questions. Cross-sectional studies were rated as high-risk (0-3 points), moderate-risk (4-6 points), and low-risk (7-9 points).

### Data Synthesis

Data from the selected studies were extracted and charted by one reviewer (CY) across four sections: (1) study characteristics (eg, first author, publication year, region, study design, and target population), (2) sample characteristics (eg, sample size, mean age, female proportion, education level, and disease presence), (3) variables and measurements (eg, cyberchondria measures and other health-related variables), and (4) QoL domains associated with cyberchondria.

A narrative analysis approach was adopted to synthesize the findings. All extracted variables were categorized according to the World Health Organization Quality of Life (WHOQOL) framework, which includes 5 domains physical health, psychological health, level of independence, social relationships, and environment [27]. Additionally, a separate behavior category was included to capture behavior-related variables that are not fully represented in the original WHOQOL domains. Each variable was assigned to the most relevant category, and the direction of association with quality of life was summarized.

### Ethical Considerations

As this study was solely literature-based and did not involve any research participants, no formal ethics approval was required.

## Results

### Overview

**Figure 1:**
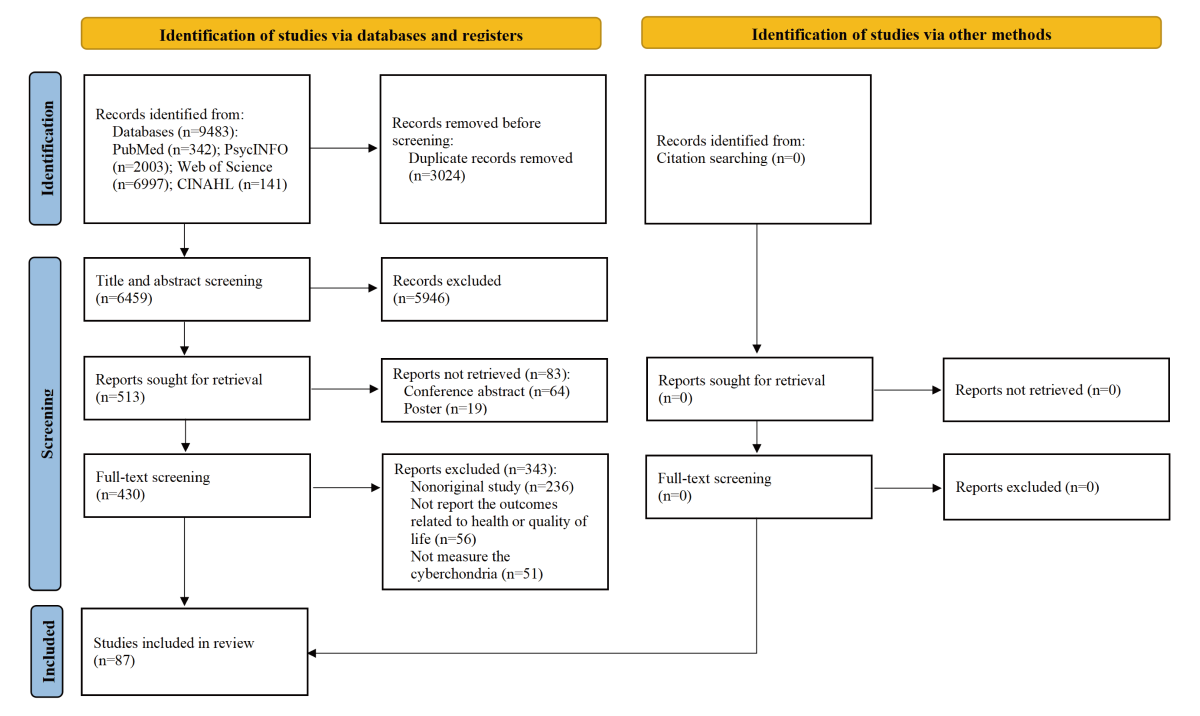
PIRSMA (Preferred Reporting Items for Systematic Reviews and Meta-Analyses) flowchart of the screening and selection process.

A total of 9483 records were identified from the four electronic databases. After removing duplicates, screening title, abstract, and full-text, 87 studies were ultimately included in this review (Figure 1) [4,5,7,28-111]. The interrater reliability was 0.85 (95% CI 0.79-0.90).

### Study Characteristics

Table 2 summarizes the characteristics of the included studies. Most of these studies (83.9%) were published within the past 6 years (2020-2025). All 87 studies used a cross-sectional design. The research spanned regions of varying income levels, as classified by the World Bank [112]. Nearly half were conducted in high-income regions (43.7%), with a similar proportion in upper-middle-income regions (40.2%). Approximately 47% of the study recruited fewer than 500 participants. Three studies did not report the recruitment criteria [32,44,54]. Three studies focused on patients with physical diseases [43,72,108], one study examined psychological diseases [99], and 2 studies addressed pregnant women [34,95]. The remaining studies did not impose restrictions on participants based on health conditions, which included general populations and individuals in various social roles, such as students (n=21) [7,28,30,33,48,52,61,62,69,71,74-76,87,91,94,97,101,109,110], teachers (n=1) [70], nurses (n=3) [37,55,79], and parents (n=1) [53]. Detailed characteristics are presented in Multimedia Appendix 3.

**Table 2:** Summary of study characteristics (N=87).

Characteristics			Studies, n (%)		References
**Publication year**					
	2014-2018	14 (16.1)		[7,39,40,43,57-62,78,82,83,91]	
	2020-2022	36 (41.4)		[4,28,29,31,32,35,36,38,42,44,46,49,50,63,66,68,70,73,76,77,80,84-87,89,90,92-94,98,99,102,105,109,111]	
	2023-2025	37 (42.5)		[5,30,33,34,37,41,45,47,48,51-56,64,65,67,69,71,72,74,75,79,81,88,95-97,100,101,103,104,106-108,110]	
**Study design**					
	Cross-sectional	87 (100)		[4,5,7,28-111]	
**Regions (income level)**					
	High-income regions	38 (43.7)		[4,7,29,32,35,36,38-43,46,51,57-61,64-68,75-78,80-84,89,95,98,99,111]	
	Upper-middle-income regions	35 (40.2)		[5,31,34,37,45,47-50,53-55,63,69-74,85,90-93,97,100-105,107-110]	
	Lower-middle-income regions	11 (12.6)		[28,30,33,52,56,79,86-88,94,96]	
**Sample size**					
	<500	42 (46.7)		[7,28,32-34,37-39,43,47-50,59-63,67,69,75,77-79,83,84,86,87,89-91,93-96,99-101,107,108]	
	500-1000	33 (36.7)		[4,5,29,35,36,40-42,45,46,51,53,54,56-58,64,65,70-72,76,80-82,85,92,97,98,102,104,106]	
	>1000	15 (16.7)		[30,31,44,52,55,66,68,73,74,88,103,105,109-111]	
**Population (health condition)**					
	General population	55 (61.1)		[4,5,29,31,35,36,38-42,45-47,49-51,56-61,63-68,73,77,78,80-86,89,90,92,93,96,98,100,102-107,111]	
	Population with physical disease	3 (3.3)		[43,72,108]	
	Population with psychological disease	1 (1.1)		[99]	
	Pregnant women	2 (2.2)		[34,95]	
**Population (social roles)**					
	Students	21 (23.3)		[7,28,30,33,48,52,61,62,69,71,74-76,87,91,94,97,101,109,110]	
	Teachers	1 (1.1)		[70]	
	Nurses	3 (3.3)		[37,55,79]	
	Parents of children attending the clinic	1 (1.1)		[53]	
**Measurement**					
	^a^CSS	37 (41.1)		[7,32,36,37,39,40,43,46,48,51,53,57-62,65,67,69-72,76-78,80-84,87,90,91,93,99,109]	
	Short version of CSS	44 (48.9)		[4,5,28,30,31,33-35,38,41,42,44,45,47,52,54-56,61,64,68,73,75,79,85,86,88,89,92,94,96-98,101-106,108,110,111]	
	^b^SCS	6 (6.7)		[29,50,66,74,95,100]	
	^c^CS	3 (3.3)		[49,63,107]	

^a^CSS: Cyberchondria Severity Scale.

^b^SCS: Short Cyberchondria Scale.

^c^CS: Cyberchondria Scale.

### Measurement of Cyberchondria

The majority of studies used the Cyberchondria Severity Scale (CSS; 41.1%) or its shorter versions (48.9%) to assess cyberchondria. The remaining 9 studies used the Short Cyberchondria Scale (SCS) [29,50,66,74,95,100] (n=6) or the Cyberchondria Scale (CS) [49,63,107] (n=3; Table 2, Multimedia Appendix 3).

### Risk-of-Bias Results

Most of the included studies were rated as having moderate (54.4%) or low risk of bias (36.7%; see Multimedia Appendix 4). All studies scored 2 points in the “Assessment of exposure and outcome” domain due to the absence of gold-standard assessment tools for evaluating cyberchondria and associated health-related outcomes. Studies classified as high risk performed poorly in both the “Study sample selection” and “Confounding factors” domains.

### Impact of Cyberchondria on QoL

All factors related to cyberchondria were classified into 6 distinct categories pertaining to QoL. The associations with psychological health were most frequently reported (Table 3, Multimedia Appendix 3).

**Table 3:** Summary of categories (quality of life domains) associated with cyberchondria.

Categories and variables			Direction		Studies, n (%)		References
**Physical health**							
	Pain and discomfort	Positive		9 (10)		[35,36,40,64,74,85,98,101,111]	
	Sleep quality	Negative		4 (4.4)		[35,98,105,110]	
	Oral health	No association		1 (1.1)		[108]	
	Temporomandibular disorders	Positive		1 (1.1)		[45]	
**Psychological health**							
	Anxiety	Positive		55 (61.1)		[4, 7, 28, 29, 32-36, 38-40, 42, 43, 45, 46, 48, 49, 56-64, 66-68, 71, 73, 75, 77, 80, 82, 85, 86, 88-91, 93-95, 97-102, 104, 105]	
	Fear	Positive		13 (14.4)		[31,44,50,54,55,64,65,84,86,92,102,106,111]	
	Intolerance of uncertainty	Positive		13 (14.4)		[4,29,36,43,44,58,60,61,64,82,87,102]	
	Negative feelings or emotions	Positive		13 (14.4)		[29,30,32,35,54,55,62,70,73,81,98,104,109]	
	Anxiety sensitivity	Positive		12 (13.3)		[28,34,58,60,61,79,82,87,91,92,101,102]	
	Obsessive-compulsive symptoms	Positive		12 (13.3)		[35,36,38,39,60,61,83,86,98,101,105]	
	Depression	Positive		9 (10.0)		[7,35,40,63,74,84,98,100,101]	
	Metacognitive beliefs	Positive		8 (8.9)		[32,54,60,61,80,81,92]	
	Stress	Positive		6 (6.7)		[7,41,50,63,86,109]	
	Death anxiety	Positive		3 (3.3)		[47,71,75]	
	Emotion dysregulation	Positive		3 (3.3)		[42,56,81]	
	Self-esteem	Negative		3 (3.3)		[35,38,98]	
	Alexithymia	Positive		2 (2.2)		[55,109]	
	Affective temperament	Positive		1 (1.1)		[84]	
	Cognitive fusion	Positive		1 (1.1)		[104]	
	Depression	Negative		1 (1.1)		[36]	
	Dispositional optimism	Negative		1 (1.1)		[76]	
	Orthorexia nervosa	Positive		1 (1.1)		[37]	
	Rumination	Positive		1 (1.1)		[41]	
	Suicidal ideation	Positive		1 (1.1)		[103]	
**Level of independence**							
	Usual or daily activities	Negative		5 (5.6)		[35,86,96,98,104]	
	Mobility	Negative		3 (3.3)		[35,98,104]	
	Self-care	Negative		3 (3.3)		[35,98,104]	
**Social relationships**							
	Personal relationship	Negative		6 (6.7)		[35,46,73,74,78,98]	
	Communication	Positive		4 (4.4)		[35,72,74,98]	
	Social support	Negative		3 (3.3)		[35,46,98]	
**Environment**							
	eHealth literacy	Positive		6 (6.7)		[51,65,71,72,85,110]	
	Financial satisfaction	Negative		4 (4.4)		[35,46,74,98]	
	Work satisfaction	Negative		4 (4.4)		[35,86,96,98]	
	Health literacy	Positive		3 (3.3)		[33,53,88]	
	Recreation opportunities	Negative		3 (3.3)		[35,46,98]	
	Living place satisfaction	Negative		2 (2.2)		[35,98]	
	eHealth literacy	No association		1 (1.1)		[69]	
**Behavior**							
	Addictive behavior	Positive		14 (15.6)		[28,30,31,35,49,51,52,79,91,97,100,101,106,107]	
	Coping strategy	Negative		1 (1.1)		[5]	
	Health care usage	Positive		1 (1.1)		[40]	
	Health promotion behavior	Positive		1 (1.1)		[66]	
	Overuse of health care	Positive		1 (1.1)		[5]	
	Problematic internet use	Positive		1 (1.1)		[92]	

#### Category 1: Physical Health

Pain and discomfort were found to be the most frequently examined physical health domains in relation to cyberchondria, with 9 studies investigating this association [35,36,40,64,74,85,98,101,111]. Four studies identified a significant relationship between higher levels of cyberchondria and poorer sleep quality [35,98,105,110]. Other health-related outcomes received less attention; for example, only one study found a positive correlation between cyberchondria and temporomandibular disorders [45], while another study reported a nonsignificant association between cyberchondria and oral health [108].

#### Category 2: Psychological Health

Anxiety was the most frequently reported psychological factor associated with cyberchondria, with more than half of the included studies (61.1%) identifying a significant correlation between them [4, 7, 28, 29, 32-36, 38-40, 42, 43, 45, 46, 48, 49, 56-64, 66-68, 71, 73, 75, 77, 80, 82, 85, 86, 88-91, 93-95, 97-102, 104, 105], highlighting the central role of anxiety in the manifestation of cyberchondria. Three studies specifically examined death anxiety and found positive correlations with cyberchondria [47,71,75] suggesting that concerns about mortality may further intensify online health-related behaviors. Nine studies reported positive correlations between cyberchondria and depression [7,35,40,63,74,84,98,100,101], while one study found a negative association [36].

A range of emotional and cognitive vulnerabilities are also important in understanding cyberchondria, including fear (n=13) [31,44,50,54,55,64,65,84,86,92,102,106,111], intolerance of uncertainty (n=13) [4,29,36,43,44,58,60,61,64,82,87,102], negative feelings or emotions (n=13) [29,30,32,35,54,55,62,70,73,81,98,104,109], anxiety sensitivity (n=12) [28,34,58,60,61,79,82,87,91,92,101,102], and obsessive-compulsive symptoms (n=12) [35,36,38,39,60,61,83,86,98,101,105].

Additionally, a few studies reported associations with metacognitive beliefs [32,54,60,61,80,81,92], stress [7,41,50,63,86,109], emotion dysregulation [42,56,81], self-esteem [35,98,104], alexithymia [55,109], affective temperament [84], cognitive fusion [104], dispositional optimism [76], orthorexia nervosa [37], rumination [41], and suicidal ideation [103].

#### Category 3: Level of Independence

Within the domain of level of independence, cyberchondria was most frequently found to be negatively associated with usual or daily activities (n=5) [35,86,96,98,104], followed by mobility (n=3) [35,98,104], and self-care (n=3) [35,98,104].

#### Category 4: Social Relationship

Disruptions in personal relationships are the most commonly studied social correlate of cyberchondria (n=6), indicating negative correlations [35,46,73,74,78,98], while positive associations with communication (n=4) [35,72,74,98], and negative associations with social support (n=3) [35,46,98].

#### Category 5: Environment

Positive associations between cyberchondria and eHealth literacy were reported most frequently (n=6) [51,65,71,72,85,110]. However, Kalantari et al [69] reported a nonsignificant correlation between cyberchondria and eHealth literacy. Satisfaction with financial status was examined in 4 studies [35,46,74,98], all reporting negative associations. Satisfaction with work was investigated in 4 studies [35,86,96,98]. Health literacy [33,53,88], recreational opportunities [35,46,98], and living place satisfaction [35,98], were also showing negative associations.

#### Category 6: Behavior

Behavior is not included in the WHOQOL framework, but it has been found to be highly associated with cyberchondria. Addictive behaviors (ie, internet addiction, social media addiction, and smartphone addiction) were the most frequently examined, with 14 studies reporting positive associations with cyberchondria [28,30,31,35,49,51,52,79,91,97,100,101,106,107]. The relationships between cyberchondria and coping strategies [5], health care usage [40], health promotion behaviors [66], overuse of health care [5], and problematic internet use [92] were also reported.

## Discussion

### Principal Findings

This scoping review synthesizes current evidence on the effects of cyberchondria across various dimensions related to QoL, including physical health, psychological health, level of independence, social relationships, environment, and behavior. Among these, psychological health emerged as the most frequently explored domain in relation to cyberchondria, with anxiety being the most commonly reported factor; more than half of the included studies identified a significant positive correlation between higher levels of cyberchondria and increased anxiety. Physical health aspects were also prominent, particularly pain and discomfort, which were examined in 9 studies as the most frequently investigated physical domains associated with cyberchondria. Additionally, 4 studies reported a significant relationship between elevated cyberchondria and poorer sleep quality. Behavior, although not formally included in the WHOQOL framework, was found to be highly associated with cyberchondria in multiple studies.

### Comparison With Prior Research and Recommendations

Despite the growing body of research on cyberchondria, the field remains in its early stages, with studies primarily focused on conceptualizing the phenomenon and developing theoretical frameworks. This emphasis on foundational work limits the depth of empirical evidence regarding cyberchondria’s mechanisms, prevalence, and broader impacts on QoL, underscoring the need for more comprehensive investigations to advance understanding and inform clinical practice [1]. Health anxiety and obsessive-compulsive features are central to the current conceptualization of cyberchondria, as evidenced by prior research [3,12,14,16] and supported by the findings of this scoping review. Research indicates the disproportionate reliance on the CSS for conceptualizing cyberchondria [113]. Although it exhibits robust psychometric properties, the internal consistency of its excessiveness and reassurance domains requires further improvement [56,66,67]. Additionally, the absence of a standardized cutoff score for the CSS hinders the interpretability of findings, particularly in distinguishing clinical from nonclinical levels of cyberchondria.

The risk of bias assessment indicated that the majority of included studies were at low or moderate risk, suggesting a generally robust body of evidence. Nonetheless, moderate risks may have influenced the findings by introducing potential overestimation of treatment effects or underreporting of adverse outcomes. Further analyses excluding studies with moderate risk of bias confirmed that the primary conclusions remained consistent, supporting the reliability of our synthesis.

The correlation between cyberchondria and domains other than psychological health has been relatively understudied. Most existing studies have focused primarily on the psychological consequences of cyberchondria, such as anxiety [7,49,63,94], depression [7,36,63], and negative feelings [74,81]. However, exploring comprehensive QoL aspects can provide a more complete understanding of how cyberchondria affects overall well-being, help identify at-risk populations, and inform the development of holistic interventions that go beyond psychological symptoms. Future studies should aim to investigate the associations between cyberchondria and a wider range of QoL domains.

Furthermore, cyberchondria itself is fundamentally characterized by excessive OHIS, which is a behaviorally driven symptom [3,12]. It is therefore not surprising that cyberchondria is associated with a range of health-related behaviors. In this review, more than ten studies reported significant associations between cyberchondria and various forms of addictive behaviors, including internet addiction, social media addiction, and smartphone addiction [28,30,31,35,49,51,52,79,91,97,100,101,106,107]. One possible explanation is that individuals with high levels of cyberchondria may engage in compulsive online searching as a maladaptive coping strategy [14,16], which shares features with behavioral addictions such as loss of control, preoccupation, and continued engagement despite negative consequences [114,115]. However, it is important to note that, currently, cyberchondria is not formally classified as a behavioral addiction, and the available evidence does not definitively establish it as such [13,116]. The observed associations between cyberchondria and various addictive behaviors indicate a potential for conceptual and clinical overlap [31,49,91]. This raises the intriguing possibility that, in the future, cyberchondria could be considered as a behavioral addiction or at least approached through interventions developed for addictive behaviors. Nevertheless, more research is needed to clarify whether cyberchondria meets the diagnostic criteria for behavioral addiction, to elucidate shared and distinct risk factors, and to evaluate the effectiveness of addiction-focused interventions for individuals with cyberchondria.

The relationship between cyberchondria and QoL is complex. Conflicting findings exist. For example, Arsenakis et al [36] reported a contrast direction of cyberchondria and depression with other related studies. This unexpected finding may stem from methodological issues of multiple regression, which isolates each predictor’s unique contribution to the dependent variable while controlling for others, thereby partitioning shared variance among highly intercorrelated independents [117,118]. Consequently, multicollinearity among the psychopathology measures induces a suppression effect, reversing the PHQ-9 coefficient’s sign to negative after accounting for overlapping variance from other predictors [119,120]. Moreover, Kalantari et al [69] reported a nonsignificant correlation when examining the total CSS score, a difference with other studies. However, it is noteworthy that specific subscales of cyberchondria, such as excessiveness and reassurance, did show significant positive correlations with eHealth literacy in the same study. This indicates that certain behavioral dimensions of cyberchondria, particularly those involving excessive searching and reassurance-seeking, are more closely linked to individuals’ abilities to access and evaluate online health information. These nuanced findings highlight the importance of examining cyberchondria at the subscale level, as associations with eHealth literacy may vary across different facets of the construct. Future research may benefit from a more granular approach, exploring how specific aspects of cyberchondria interact with digital health competencies. Additionally, cyberchondria may not only directly affect QoL domains but also play a mediating or moderating role between other psychological or behavioral factors and QoL outcomes. For example, psychological distress could increase cyberchondria severity, which then impacts sleep quality [105] and death anxiety [75]. The relationship may also be bidirectional. A higher level of cyberchondria could lead to addictive internet usage [35], stress [86], or psychological insecurity [74], which in turn further reduces QoL. These complex and potentially cyclical links highlight the need for future research to use mediation and moderation analyses and to consider factors such as digital literacy. Understanding these dynamics is important for developing targeted interventions and for gaining a clearer picture of the overall impact of cyberchondria.

### Limitations

First, all included studies used cross-sectional designs. The absence of randomized controlled trials and longitudinal studies limits the ability to establish causal relationships between cyberchondria and QoL. Cross-sectional data potentially confound the temporal dynamics and directionality of cyberchondria’s impact on QoL. Second, the limited focus of included studies on participants from lower-income regions. This geographic and socioeconomic bias may limit the generalizability of findings, as cyberchondria’s prevalence, manifestations, and impact on QoL may vary across diverse cultural, economic, and health care contexts. Third, there is the limited number of studies examining the relationship between cyberchondria and QoL domains other than psychological health. Forth, this review excluded studies that used health anxiety as a direct measure of cyberchondria. While this approach was taken to maintain conceptual integrity and to ensure that the included studies specifically assessed cyberchondria rather than its overlapping constructs, it may have influenced the scope and findings of the review. Health anxiety is widely recognized as a core feature of cyberchondria, and excluding studies that equate the 2 could mean that some relevant research was omitted, potentially narrowing the range of evidence considered. This limitation highlights the ongoing challenges in distinguishing cyberchondria from related psychological constructs.

### Conclusion

This scoping review demonstrates the primary risk factors and challenges influencing the multidimensional QoL in individuals with cyberchondria, consisting of physical, psychological, social relationship, level of independence, environment, and behavior. Psychologists serve as vital mediators in these domains. The findings have implications for clinical practice, indicating the imperative for clinicians to adopt a comprehensive approach in evaluating and managing cyberchondria, thereby mitigating its wide-ranging effects on QoL through integrated interventions that address both emotional and behavioral components. Additionally, the results highlight the urgent need to address problematic and addictive patterns of internet use that may exacerbate cyberchondria and negatively impact overall well-being. Developing evidence-based guidelines to identify and manage internet-related addictive behaviors is essential to mitigate their harmful effects and prevent unnecessary strain on health care systems. For future research, longitudinal and randomized controlled trials are recommended to establish causal relationships, while expanding studies to underrepresented geographic and socioeconomic contexts could enhance generalizability and inform culturally tailored intervention strategies.

## Appendix

Multimedia Appendix 1 PRISMA checklist.

Multimedia Appendix 2 Search strategies.

Multimedia Appendix 3 Data extraction chart.

Multimedia Appendix 4 Risk of bias assessment.

## Abbreviations

CS: Cyberchondria Scale
CSS: Cyberchondria Severity Scale
NOS-xs: Newcastle-Ottawa Scale for cross-sectional study
OHIS: online health information seeking
PICO: Participants, Interventions, Comparisons, and Outcomes
PRISMA-ScR: Preferred Reporting Items for Systematic reviews and Meta-Analyses extension for Scoping Reviews
QoL: quality of life
SCS: Short Cyberchondria Scale
WHOQOL: World Health Organization Quality of Life

## Data Availability

The datasets generated and/or analyzed during the study are available from the corresponding author.
