# Supplementary material for: Impact of Cyberchondria on Health and Quality of Life: Scoping Review
Source: J Med Internet Res. 2025 Dec 4;27:e77977. doi: 10.2196/77977 (PMC12715475; doi:10.2196/77977)
Supplement: Multimedia Appendix 2 [file jmir_v27i1e77977_app2.docx]

**Appendix 1. Search Strategy from inception to October 9, 2025**

| **#** | **Searches** | **Results** |
| --- | --- | --- |
| **Database: Pubmed** | | |
| 1 | "Cyberchondria*"[Title/Abstract] | 222 |
| 2 | "Cyber"[Title/Abstract] OR "Internet"[Title/Abstract] OR "Web-based"[Title/Abstract] OR "Online"[Title/Abstract] OR "Web"[Title/Abstract] | 609,635 |
| 3 | "Search*"[Title/Abstract] OR "Seek*"[Title/Abstract] OR "Check*"[Title/Abstract] OR "Brows*"[Title/Abstract] OR "Reassur*"[Title/Abstract] | 1,334,217 |
| 4 | "Hypochondriasis"[MeSH Terms] OR "hypochondria*"[Title/Abstract] OR "health anxiety"[Title/Abstract] OR "illness anxiety"[Title/Abstract] | 6,487 |
| 5 | #2 AND #3 AND #4 | 202 |
| 6 | #1 OR #5 | 342 |
| **Database: APA PsycINFO** | | |
| 1 | tiab(Cyberchondria*) | 138 |
| 2 | tiab(Cyber) OR tiab (Internet) OR tiab (Web-based) OR tiab (Online) OR tiab (Web) | 227,660 |
| 3 | tiab(Search*) OR tiab (Seek*) OR tiab(Check*) OR tiab(Brows*) OR tiab(Reassur*) | 358,298 |
| 4 | tiab(Hypochondria*) OR tiab(Health anxiety) OR tiab(Illness anxiety) | 71,658 |
| 5 | [S2] AND [S3] AND [S4] | 1,934 |
| 6 | [S1] OR [S5] | 2,003 |
| **Database: Web of Science** | | |
| 1 | Cyberchondria* (Topic) | 400 |
| 2 | Cyber (Topic) or Internet (Topic) or Web-based (Topic) or Online (Topic) or Web (Topic) | 1,742,204 |
| 3 | Search* (Topic) or Seek* (Topic) or Check* (Topic) or Brows* (Topic) or Reassur* (Topic) | 2,711,172 |
| 4 | Hypochondria* (Topic) or Health anxiety (Topic) or Illness anxiety (Topic) | 187,348 |
| 5 | #2 AND #3 AND #4 | 6,813 |
| 6 | #1 OR #5 | 6,997 |
| **Database: CINAHL Plus** | | |
| 1 | AB Cyberchondria* | 91 |
| 2 | AB Cyber OR AB Internet OR AB Web-based OR AB Online OR AB Web | 178,209 |
| 3 | AB Search* OR AB Seek* OR AB Check* OR AB Brows* OR AB Reassur* | 348,292 |
| 4 | AB Hypochondria* OR AB Health anxiety OR AB Illness anxiety | 9,953 |
| 5 | S2 AND S3 AND S4 | 123 |
| 6 | S1 OR S5 | 141 |
