# Supplementary material for: Impact of Cyberchondria on Health and Quality of Life: Scoping Review
Source: J Med Internet Res. 2025 Dec 4;27:e77977. doi: 10.2196/77977 (PMC12715475; doi:10.2196/77977)
Supplement: Multimedia Appendix 3 [file jmir_v27i1e77977_app3.docx]

**Appendix 2. Data extraction chart**

| **First author, year** | **Region** | **Study design** | **Targeted population** | **Sample characteristics** | | | | | **Measurement tool for CYB** | **Other health-related valuables (measurement tool)** | **Categories associated with cyberchondria** |
| --- | --- | --- | --- | --- | --- | --- | --- | --- | --- | --- | --- |
|  |  |  |  | **Sample size (n)** | **Mean age (SD)**  **[Range]** | **Female proportion (%)** | **Education level/years (SD)** | **Medical condition** |  |  |  |
| Fergus, 2014[1] | United States | Cross-sectional study | General population | 539 | 31.3 (9.9)  [18-67] | 43.4 | Tertiary or above: 59.6% | NR | CSS | Health Anxiety, HA (SHAI) | CYB positively associates with HA, AS, and IU |
| McElroy & Shevlin, 2014[2] | United Kingdom | Cross-sectional study | Undergraduate students | 208 | 24.2 (8.2)  [18-60] | 63.9 | Tertiary or above: 100% | NR | CSS | Anxiety (DASS-21)  Depression (DASS-21)  Stress (DASS-21) | CYB positively associates with anxiety, depression, and stress |
| Fergus, 2015[3] | United States | Cross-sectional study | General population (over 18 years of age) | 578 | 31.2 (9.8) | 43.7 | Tertiary or above: 59.6% | NR | CSS | Anxiety Sensitivity, AS (ASI-3)  Intolerance of Uncertainty, IU (IUS-12)  Health Anxiety, HA (WI-6) | AS positively associates with CYB |
| Norr et al., 2015a[4] | United States | Cross-sectional study | General population (over 18 years of age) | 526 | 34.9 (12.4)  [18-72] | NR | Tertiary or above: 35.2% | NR | CSS | Anxiety Sensitivity, AS (ASI-3)  Health Anxiety, HA (SHAI)  Intolerance of Uncertainty, IU (IUS-12) | AS and IU are the potential risk factors of CYB |
| Norr et al., 2015b[5] | United States | Cross-sectional study | General population (over 18 years of age) | 468 | 35.4 (12.5)  [18-72] | 71.6 | Tertiary or above: 37.4% | Obsessive-compulsive symptoms: 20.5%  Health anxiety: 15.0% | CSS | Obsessive-Compulsive Symptoms, OCS (DOCS) | CYB positively associates with OCS |
| Barke et al., 2016[6] | Germany | Cross-sectional study | General population (over 18 years of age) | 500 | 29.1 (10.4) | 73.6 | NR | NR | CSS | Depression (CES-D)  Health Anxiety, HA (SHAI)  Health-care Utilization, HCU (HCU)  Somatic Symptoms, SS (PHQ-15) | CYB positively associates with HA, HCU, SS, depressive symptoms. |
| Fergus & Russell, 2016[7] | United States | Cross-sectional study | General population (over 18 years of age) | 375 | 31.6 (10.2)  [19-64] | 47.3 | Tertiary or above: 60.1% | NR | CSS | Health Anxiety, HA (MIHT)  Obsessive-Compulsive Symptoms, OCS (DOCS) | CYB positively associates with HA |
| Fergus & Spada, 2017[8] | United States | Cross-sectional study | General population (over 18 years of age) | 260 | 32.9 (9.2)  [18-65] | 40.8 | Tertiary or above: 69.1% | Diagnosed with physical health problems: 25.5% | CSS | Anxiety Sensitivity, AS (ASI-3)  Health Anxiety, HA (WI-6)  Intolerance of Uncertainty, IU (IUS-12)  Metacognitive Beliefs, MB (MCQ-HA) | CYB positively associates with HA, IU, MB and AS |
| Fergus & Spada, 2018a[9] | United States | Cross-sectional study | Undergraduate students | 330 | 19.4 (2.1)  [18-47] | 66.6 | Tertiary or above: 100% | NR | CSS | Anxiety Sensitivity, AS (ASI-3)  Health Anxiety, HA (WI-6)  Uncertainty, IU (IUS-12)  Metacognitive Beliefs, MB (MCQ-HA)  Obsessive-Compulsive Symptoms, OCS (BARI & SSQ) | CYB positively associates with HA, IU, MB, AS, and OCS |
| Fergus & Spada, 2018b[9] | United States | Cross-sectional study | General population (over 18 years of age) | 331 | 38.7 (10.4)  [22-74] | 68.6 | Tertiary or above: 68.3% | NR | CSS-SF (15 items) | Health Anxiety, HA (WI-6)  Metacognitive Beliefs, MB (MCQ-HA)  Obsessive-Compulsive Symptoms, OCS (DOCS & BARI & SSQ) | CYB positively associates with HA, MB, and OCS |
| Mathes et al., 2018[10] | United States | Cross-sectional study | General population (over 18 years of age) | 462 | 36.6 (12.9)  [18-77] | 64.3 | Tertiary or above: 71% | NR | CSS | Disability in Social Functioning, DSF (SDS)  Quality of Life, QOL (WHOQOL) | CYB → DSF (positive)  Nonsignificant associations between CYB, QOL |
| Selvi et al., 2018[11] | Turkey | Cross-sectional study | University students | 337 | 21.8 (5.2)  [16-55] | 55.8 | Tertiary or above: 100% | NR | CSS | Anxiety Sensitivity, AS (ASI-3)  Health Anxiety, HA (SHAI)  Internet Addiction, IA (IAT) | CYB positively associate with AS, IA, and HA |
| Bajcar et al., 2019[12] | Poland | Cross-sectional study | General population (over 18 years of age) | 380 | 26.5 (11.1)  [19-68] | 53.4 | Tertiary or above: 31% | NR | CSS | Obsessive-Compulsive Symptoms, OCS (DOCS)  Health Anxiety, HA (SHAI) | CYB positively associate with HA and OCS |
| Blackburn et al., 2019[13] | United States | Cross-sectional study | Orthopaedic patients | 104 | 49.0 (15.0) | 59.0 | 16 (2.9) | Orthopaedic disease (100%)  Multiple pain (37%) | CSS | Health Anxiety, HA (SHAI)  Intolerance of Uncertainty, IU (IUS-12) | IU → CYB → HA  CYB positively associate with IU and HA |
| Gibler et al., 2019[14] | NR | Cross-sectional study | University students | 221 | 19.2 (1.7)  [18-33] | 70.6 | Tertiary or above: 100% | Chronic pain: 20.8% | CSS | Health Anxiety, HA (SHAI)  Pain Catastrophizing, PC (PCS^1^) | HA → CYB (positive)  PC → CYB (positive) |
| Jokic-Begic et al., 2020[15] | Croatia | Cross-sectional study | General population | 1813 | 18-77 | 80.6 | Tertiary or above: 74.7% | Chronic conditions: 13.4% | SCS | Coronavirus Anxiety, CA (CAS)  COVID-19 Safety Behaviour, CSB (CSBS) | CYB positively associate with CAS and CSB |
| Jungmann & Witthöft, 2020[16] | Germany | Cross-sectional study | General population | 1615 | 33.4 (13.2)  [16-81] | 79.8 | NR | NR | CSS-SF (12 items) | Coronavirus Anxiety, CA (Questions specific to the COVID-19 pandemic)  Health Anxiety, HA (SHAI) | CYB positively associate with CA (Moderator: HA) |
| Maftei & Holman, 2020[17] | Romania | Cross-sectional study | University students | 823 | 34.3 (10.2)  [15-67] | 64.8 | Tertiary or above: 48.6% | NR | CSS | Dispositional Optimism, DO (LOT)  Psychological distress, PD (The Neuroticism Scale) | CYB associates with PD positively, and DO negatively |
| Marino et al., 2020[18] | Italy | Cross-sectional study | General population (over 18 years of age) | 343 | 25.8 (5.3)  [18-59] | 87.0 | Tertiary or above: 76.4% | NR | CSS | Health Anxiety, HA (HAQ) | CYB positively associate with HA |
| Seyed et al., 2020[19] | Iran | Cross-sectional study | General population | 651 | 33.5 (10.8)  [13-73] | 62.4 | Tertiary or above: 66.9% | NR | CSS-SF (12 items) | Anxiety Sensitivity, AS (ASQ)  Fear of COVID-19, FCV (FCV-19S)  Metacognitive Beliefs, MB (MCQ-30)  Problematic Internet Use, PIU (GPIUS) | PIU → CYB (positive)  CYB → AS/FCV/MB (positive) |
| Shailaja et al., 2020[20] | India | Cross-sectional study | University students | 300 | 22.6 (2.9)  [18-37] | 82.0 | Tertiary or above: 100% | NR | CSS-SF (15 items) | Anxiety (DASS-21) | CYB positively associates with anxiety |
| Arsenakis et al., 2021[21] | Switzerland | Cross-sectional study | General population (over 18 years of age) | 749 | 34.0  [18-75] | 67.6 | NR | NR | CSS | Somatic Symptoms, SS (PHQ-15)  Depression (PHQ-9)  Health Anxiety, HA (SHAI)  Intolerance of Uncertainty, IU (IUS-12)  Obsessive-Compulsive symptoms, OCS (OCI-R) | SS/ HA/IU/OCS → CYB (positive)  CYB associates with depression negatively |
| Bajcar & Babiak, 2021[22] | Poland | Cross-sectional study | General population (over 18 years of age) | 207 | 31.5 (13.02)  [19-64] | 58.9 | NR | NR | CSS-SF (30 items) | Health Anxiety, HA (SHAI)  Obsessive-Compulsive Symptoms, OCS (DOCS)  Self-esteem (RSES) | CYB → HA/OCS → Self-esteem  CYB positively associate with HA and OCS, negatively associate with self-esteem |
| Durak Batıgün et al., 2021[23] | Turkey | Cross-sectional study | General population | 442 | 36.5 (13.8)  [18-65] | 55.1 | NR | NR | CS | Anxiety (BSI)  Internet Addiction, IA (IAT)  Health Anxiety, HA (SHAI) | AS→IA→CYB  HA→IA→CYB  CYB positively associate with AS, IA, and HA |
| Han et al., 2021[24] | China | Cross-sectional study | General population | 486 | 22.9 (5.7)  [14-50] | 71.8 | NR | NR | CS | Anxiety (DASS-21)  Depression (DASS-21) | CYB → Anxiety/ Depression (positive) |
| Oniszczenko, 2021[25] | Poland | Cross-sectional study | General population (over 18 years of age) | 499 | 29.3 (9.8)  [18-72] | 87.2 | Tertiary or above: 60.7% | NR | CSS | Anxious Temperament, AT (TEMPS-A)  Depressive Temperament, DT (TEMPS-A)  Fear of COVID-19, FCV (FCV-19S) | AT → FCV → CYB  CYB positively associate with AT, DT and FCV |
| Peng et al., 2021[26] | China | Cross-sectional study | General population | 674 | [16-70] | 56.4 | Tertiary or above: 65.2% | Chronic conditions: 20.3% | CSS-SF (12 items) | e-Health Literacy, eHL (eHEALS)  Health Anxiety, HA (SHAI)  Somatic Symptoms, SS (PHQ-15) | CYB positively associate with eHL, HA, and SS |
| Rahme et al., 2021[27] | Lebanon | Cross-sectional study | General population (over 18 years of age) | 499 | 24.3 (8.2) | 70.6 | Tertiary or above: 78.8% | NR | CSS-SF (12 items) | Anxiety (Lebanese anxiety scale)  Stress (Beirut distress scale)  Fear of COVID-19, FCV (FCV-19S)  Obsessive-Compulsive Symptoms, OCS (Y-BOCS)  Physical Quality of Life, PQOL (SF-12)  Mental Quality of Life, MQOL (SF-12) | CYB → Stress/FCV/OCS → PQOL  CYB → Anxiety/Stress/FCV → MQOL  CYB positively associate with anxiety, stress, FCV, and OCS |
| Sarigedik & Olmez, 2021[28] | Turkey | Cross-sectional study | General population | 407 | 29.5 (10.4)  [18-65] | 63.9 | NR | NR | CSS | Coronavirus Anxiety, CA (CAS) | CYB positively associates with CA |
| Vismara et al., 2021[29] | Italy | Cross-sectional study | Adults (18-70yrs) with a diagnosis of OCD, MDD, or ADs (panic disorder, general anxiety disorder,  social anxiety disorder) | 77 | 46.4 (12.8) | 62.3 | Tertiary or above: 32.5% | NR | CSS | Anxiety (GAD-7)  Health Anxiety, HA (HAQ)  Obsessive-Compulsive Severity, OCS (OCI-R) | CSS positively associates with HA, OCS, and Anxiety |
| Wu et al., 2021[30] | Iran | Cross-sectional study | General population without COVID-19 (over 18 years of age) | 694 | 27.9 (5.2)  [19-41] | 50.6 | NR | NR | CSS-SF (12 items) | Anxiety Sensitivity, AS (ASI-3)  Coronavirus Anxiety, CA (CAS)  Fear of COVID-19, FCV (FCV-19S)  Intolerance of Uncertainty, IU (IUS-12) | CA/FCV → AS/IU → CYB  CYB → IU → CA/FCV  CSS positively associates with AS, CA, FCV, and IU |
| Abu Khait et al., 2022[31] | Jordan | Cross-sectional study | University students | 143 | 21.2 (0.7) | 70.6 | Tertiary or above: 100% | NR | CSS-SF (8 items) | Anxiety Sensitivity, AS (ASI-3)  Health Anxiety, HA (HAI)  Internet Addiction, IA (IAT) | CSS positively associates with AS, HA and IA |
| Afrin & Prybutok, 2022[32] | United States | Cross-sectional study | General population | 533 | NR | 41.3 | Tertiary or above: 95.8% | NR | SCS | Affective Responses, AR  Health Anxiety, HA (WI-6)  Intolerance of Uncertainty, IU (IUS-12) | IU → AR → CYB  IU → HA → AR → CYB  CSS positively associates with AR, HA and IU |
| Ahorsu et al., 2022[33] | Iran | Cross-sectional study | General population | 10843 | 35.5 (12.0) | 62.3 | Tertiary or above: 64.5% | NR | CSS-SF (12 items) | Fear of COVID-19, FCV (FCV-19S)  Social Media Addiction, SMA (BSMAS)  Vaccination Intentionality, VI (ICVVS) | CSS positively associates with FCV, SMA, and VI; |
| Airoldi et al., 2022[34] | United Kingdom | Cross-sectional study | NR | 125 | 34.5 (14.1)  [19-68] | 58.4 | NR | NR | CSS | Health Anxiety, HA (SHAI)  Health Dysfunctional Beliefs, HDB (HCQ)  Metacognitive Beliefs, MB (MCQ-HA) | CSS positively associates with HA, HDB, and MB |
| Ambrosini et al., 2022[35] | Italy | Cross-sectional study | General population (over 18 years of age) | 572 | 33.6 (14.6)  [18-77] | 65.6 | NR | Physical disease (26.7%);  Psychiatric disease (7.3%) | CSS-SF (12 items) | Anxiety (HADS-A)  Depression (HADS-D)  Coronavirus Anxiety, CA (CAS)  Health Anxiety, HA (SHAI)  Internet Addiction, IA (IAT)  Obsessive-Compulsive Symptoms, OCS (MOCQ-R)  Quality of Life, QOL (WHOQOL) | CYB → Anxiety/ HA (positive) (Moderator: CA)  CYB → OCS/IA → Anxiety/ Depression/ HA/ QOL  CYB positively associates with Anxiety, Depression, HA, IA, and OCS, and negatively associates with QOL |
| Błachnio et al., 2022[36] | Poland | Cross-sectional study | General population | 615 | 43.9 (14.6)  [18-83] | 53 | NR | NR | CSS-SF (12 items) | Emotion Dysregulation, ED (DERS)  Health Anxiety, HA (SHAI)  Psychological Distress, PD (K-6) | CYB positively associates with ED, HA and PD  HA → ED → CYB |
| Bottesi et al., 2022a[37] | Italy | Cross-sectional study | General population | 556 | 29.6 (13.2) | 69.3 | NR | NR | CSS-SF (24 items) | Psychological Distress, PD (DASS-21)  Health Anxiety, HA (HAQ)  Intolerance of Uncertainty, IU (IUS-12) | IU → CYB → HA → PD  CYB positively associates with HA, IU and PD |
| Bottesi et al., 2022b[37] | Italy | Cross-sectional study | General population | 575 | 31.9 (13.4) | 31.9 | NR | NR | CSS-SF (24 items) | Psychological Distress, PD (DASS-21)  Health Anxiety, HA (HAQ)  Intolerance of Uncertainty, IU (IUS-12) | IU → CYB → PD  IU → CYB → HA → PD  CYB positively associates with HA, IU and PD |
| Boysan et al., 2022[38] | NR | Cross-sectional study | NR | 1049 | 25.4 (7.5)  [18-62] | 64.3 | NR | NR | CSS-SF (12 items) | Intolerance of Uncertainty, IU (IUS-12)  Fear of COVID-19, FCV (FCV-19S) | IU → FCV → CYB  CYB positively associates with IU and FCV |
| Ciułkowicz et al., 2022[39] | Poland | Cross-sectional study | General population | 538 | 36.7 (12.6) | 81.4 | NR | NR | CSS | Health Anxiety, HA (SHAI)  Perceived Social Support, PSS (MSPSS)  Quality of Life, QOL (QOLS) | CYB positively associates with HA, negatively associates with PSS and QOL |
| Durmuş et al., 2022[40] | Turkey | Cross-sectional study | General population (over 18 years of age) | 425 | NR | 63.3 | Tertiary or above: 56.7% | NR | SCS | Fear of COVID-19, FCV (FCV-19S)  Perceived Stress, PS (PSS) | CYB positively associates with FCV and PS |
| Karakaş et al., 2022[41] | Turkey | Cross-sectional study | Teachers | 1000 | NR | 61.7 | Tertiary or above: 100% | Chronic conditions: 57.6% | CSS | Mental Well-being, MW (WHO-5) | CYB Negatively associates with MW |
| Liu et al., 2022[42] | China | Cross-sectional study | Teenagers | 2074 | 15.8 (1.79)  [12-20] | 49.4 | NR | NR | CSS-SF (12 items) | Family Dysfunction, FD (FAD)  Health anxiety, HA (SHAI) | CYB positively associates with FD and HA  FD → HA → CYB |
| Nadeem et al., 2022[43] | United States | Cross-sectional study | General population (over 18 years of age) | 500 | [20-50] | 51.2 | Tertiary or above: 91.6% | NR | CSS | Health anxiety, HA (SHAI)  Metacognitive Beliefs, MB (MCQ-HA) | CYB associates with HA and MB; MB moderates the correlation between CYB and HA |
| Rashid et al., 2022[44] | Pakistan | Cross-sectional study | University students | 300 | 20  [18-25] | 53.0 | Tertiary or above: 100% | NR | CSS | Intolerance of Uncertainty, IU (IUS-12)  Anxiety Sensitivity, AS (ASI-3) | CYB positively associates with AS and IU |
| Santoro et al., 2022[45] | Italy | Cross-sectional study | General population (over 18 years of age) | 431 | 34.6 (12.0)  [18-74] | 63.3 | 16.5 (2.9) | Physical disease: 47.2% | CSS-SF (12 items) | Health Anxiety, HA (WI)  Somatic Symptom, SS (Level 2—Somatic Symptom—Adult Patient) | SS → HA → CYB  CYB positively associates with HA and SS |
| Sezer et al., 2022[46] | Turkey | Cross-sectional study | Women | 422 | 33.7 (10.1)  [18-49] | 100.0 | NR | NR | CSS | Anxiety (State-trait Anxiety Inventory)  Mental Well-being, MW (PWBS) | CYB positively associates with anxiety levels and MW |
| Vismara et al., 2022[47] | Italy | Cross-sectional study | General population | 572 | 33.6 (14.6) | 65.6 | Tertiary or above: 26.8% | Physical disease (26.7%);  Psychiatric disease (7.3%) | CSS-SF (12 items) | Anxiety (HADS-A)  Depression (HADS-D)  Coronavirus Anxiety, CA (CAS)  Health Anxiety, HA (SHAI)  Obsessive-Compulsive Symptoms, OCS (MOCQ)  Quality of Life, QOL (WHOQOL)  Self-esteem (RSES) | CYB positively associates with anxiety, depression, CA, HA, OCS  CYB negatively associates with self-esteem, positively associates with QOL |
| Yalçın et al., 2022[48] | Turkey | Cross-sectional study | General population (over 18 years of age) | 8276 | 39.9 (13.1)  [18-65] | 47.3 | Tertiary or above: 30.1% | Chronic disease: 8.6% | CSS-SF (12 items) | Health Anxiety, HA (SHAI)  Psychological Distress, PD (DASS-21)  Obsessive-Compulsive Symptoms, OCS (OCI-R)  Sleep Quality, SQ (PSQI) | PD → CYB → OCS/SQ  CYB positively associates with PD and OCS, and negatively associates with SQ |
| Zhou et al., 2022[49] | China | Cross-sectional study | University students | 1117 | 20.0 (1.3) | 48.0 | Tertiary or above: 100% | NR | CSS | Alexithymia (TAS-20)  Stress (DASS-21) | Alexithymia and stress positively associate with CYB |
| Zolotareva, 2022[50] | Russia | Cross-sectional study | General population (over 18 years of age) | 2011 | 40.9 (10.6)  [18-80] | 66.4 | NR | NR | CSS-SF (12 items) | Fear of COVID-19, FCV (FCV-19S)  Somatic Symptom Burden, SSB (SSS-8) | FCV → CYB → SSB  CYB positively associates with FCV and SSB |
| Błachnio et al., 2023[51] | Poland | Cross-sectional study | General population | 615 | 43.9 (14.6)  [18-83] | 53 | NR | NR | CSS-SF (12 items) | Rumination (The Dissipation–Rumination Scale)  Stress (Stress Appraisal Questionnaire) | Stress → Rumination→ CYB (positive) |
| El-Zayat et al., 2023[52] | Saudi Arabia | Cross-sectional study | General population (over 18 years of age) | 518 | 33.0 (14.0)  [18-70] | 64.1 | Tertiary or above: 83.6% | Chronic illness: 36.3% | CSS | e-Health Literacy, eHL (eHEALS)  Smartphone addiction, SA (SAS) | CYB positively associates with eHL and SA |
| Eşkisu et al., 2023[53] | Turkey | Cross-sectional study | NR | 651 | 26.1 (8.4) | 65.0 | NR | Chronic illness: 8.3% | CSS-SF (12 items) | Fear of COVID-19, FCV (FCV-19S)  Health Dysfunctional Beliefs, HDB (HCQ)  Metacognitive Beliefs, MB (MCQ-HA) | MB/FCV → HDB → CYB (positive) |
| Infanti et al., 2023[54] | France, Switzerland, Belgium, and Luxembourg | Cross-sectional study | General population | 725 | 33.3 (12.9)  [18-77] | 57.4 | Tertiary or above: 82.8% | NR | CSS-SF (12 items) | COVID-19–related Fears, CF (MAC-RF)  Health Anxiety, HA (SHAI)  Intolerance of Uncertainty, IU (IUS-12)  Social Relationship, SR (RQ)  Somatic Symptoms, SS (PHQ-15) | CYB associates with CF, HA, SS and SR |
| Jeong et al., 2023[55] | Korea | Cross-sectional study | General population (over 18 years of age) | 1000 | 43.7 (12.7)  [19-65] | 49.3 | NR | NR | CSS | Health Behavior, HB (ECBID)  e-Health Literacy, eHL (eHEALS)  Fear of COVID-19, FCV (FCV-19S) | CYB positively associates with eHL and FCV, negatively associates with HB |
| Liu et al., 2023[56] | China | Cross-sectional study | Middle school and college students | 1108 | 16.8 (3.4) | 67.5 | Tertiary or above: 36.8% | NR | SCS | Depression (ESDS)  Psychological Insecurity, PI (SQ)  Quality of Life, QOL (SWS) | CYB → PI → QOL/Depression  CYB positively associates with depression and PI, negatively associates with QOL |
| Nasiri et al., 2023[57] | Iran | Cross-sectional study | General population (over 18 years of age) | 703 | 34.1 (10.7) | 56.2 | Tertiary or above: 76.7% | NR | CSS | Health Dysfunctional Beliefs, HDB (HCQ)  Metacognitive Beliefs, MB (MCQ-HA)  Emotion Dysregulation, ED (DERS) | Personality traits → MB/HDB/ED → CYB  CYB positively associates with ED, MB, and HDB |
| Tarabay et al., 2023[58] | Lebanon | Cross-sectional study | General population | 449 | 24.3 (8.2) | 70.6 | Tertiary or above: 78.8% | NR | CSS-SF (12 items) | Quality of Life, QOL (SF-12) | CYB negatively associates with QOL |
| Varer Akpinar et al., 2023[59] | Turkey | Cross-sectional study | University students | 843 | 20.8 (1.72)  [17-29] | 61.2 | Tertiary or above: 100% | Chronic illness: 6.8% | CSS-SF (12 items) | Internet Addiction, IA (YIAT-SF)  Coronavirus Anxiety (CAS) | IA → CA → CYB (positive) |
| Vujić et al., 2023[60] | Serbia | Cross-sectional study | General population (over 18 years of age) | 471 | 38.7 (10.4) | 55.2 | Tertiary or above: 70.3% | Chronic illness: 0 | SCS | Anxiety/Depression (DASS-21)  Smartphone addiction, SA (SABAS) | CYB positively associates with anxiety, depression, and SA |
| Wang et al., 2023a[61] | China | Cross-sectional study | Undergraduate students | 220 | 18.5 (0.9)  [16-22] | 56.8 | Tertiary or above: 100% | NR | CSS-SF (18 items) | Health Anxiety, HA (SHAI)  Obsessive-Compulsive Symptoms, OCS (PI) | CYB positively associates with HA and OCS |
| Wang et al., 2023b[61] | China | Cross-sectional study | Undergraduate students | 198 | 18.9 (0.8)  [17-22] | 57.1 | Tertiary or above: 100% | NR | CSS-SF (18 items) | Anxiety Sensitivity, AS (ASI-3)  Depression (BDI-II)  Mental Well-being, MW (SDS)  Internet Addiction, IA (IAT)  Somatic Symptoms, SS (PHQ-15) | CYB positively associates with AS, SS, depression, and IA, negatively associates with MW |
| Yam et al., 2023[62] | NR | Cross-sectional study | General population | 520 | 28.6 (10.6)  [17-65] | 64.4 | NR | NR | CSS-SF (12 items) | Fear of COVID-19, FCV (FCV-19S)  Smartphone addiction, SA (SAS) | CYB positively associates with FCV and SA |
| Zhu et al., 2023[63] | China | Cross-sectional study | University students | 3015 | 20.1 (2.4)  [18-30] | 69.2 | Tertiary or above: 100% | NR | CSS-SF (12 items) | e-Health Literacy, eHL (eHEALS)  Sleep quality, SQ (PSQI) | SQ and eHL positively associates with CYB |
| Agrawal et al., 2024[64] | India | Cross-sectional study | Undergraduate students | 1041 | 20.7 (1.8) | 53.1 | Tertiary or above: 100% | NR | CSS-SF (12 items) | Smartphone addiction, SA (SAS)  Mental Well-being, MW (WHO-5) | SA positively associates with CYB, MW negatively associates with CYB |
| Ali et al., 2024[65] | Egypt | Cross-sectional study | University students (over 18 years of age) | 475 | 19-25: 79.6% | 82.5 | Tertiary or above: 100% | Chronic illness: 16.2% | CSS-SF (12 items) | Health Literacy, HL (Nursing students’ health-literacy questionnaire)  Health Anxiety, HA (WI) | CYB positively associates with HA and HL |
| Atsizata & Sögüt, 2024[66] | Turkey | Cross-sectional study | Nurses | 399 | 29.2 (6.4) | 75.4 | Tertiary or above: 95.8% | Chronic illness: 19.5% | CSS | Orthorexia Nervosa, ON (ORTO-11) | CYB positively associates with ON level |
| El-Zoghby et al., 2024[67] | Egypt | Cross-sectional study | Undergraduate students | 1435 | 21.0 (2.0) | 51.4 | Tertiary or above: 100% | NR | CSS-SF (12 items) | Smartphone addiction, SA (SAS) | CYB positively associates with SA |
| Fang et al., 2024[68] | China | Cross-sectional study | Nurses | 7617 | [20-70] | 93.4 | Tertiary or above: 95.8% | NR | CSS-SF (12 items) | Fear of COVID-19, FCV (FCV-19S)  Alexithymia (TAS-20)  Psychological Distress, PD (K-6) | CYB positively associates with FCV, Alexithymia, and PD |
| Fang & Mushtaque, 2024[69] | Pakistan | Cross-sectional study | General population (over 18 years of age) | 755 | ≥65: 10.2% | 40.2  (Transgender female: 15.8%) | Tertiary or above: 30.5% | NR | CSS-SF (12 items) | Emotion Regulation, ER (DERS)  Health Anxiety, HA (SHAI) | HA → ER → CYB  CYB positively associates with HA, and negatively associates with ER |
| Jungmann et al., 2024[70] | Germany | Cross-sectional study | Patients and general population (over 18 years of age) | 171 | Hypochondriasis group: 38.2 (11.0)  Clinical group: 36.5 (10.3)  Healthy group: 35.8 (12.3) | Hypochondriasis group: 29.0  Clinical group: 52.0  Healthy group: 33.0 | Hypochondriasis group: 11.3 (1.0)  Clinical group: 11.6 (1.0)  Healthy group: 11.8 (0.7) | NR | CSS | Health Anxiety, HA (SHAI) | CYB positively associates with HA |
| Kalantari et al., 2024[71] | Iran | Cross-sectional study | Undergraduate students | 241 | 18-24: 95.8% | 60.0 | Tertiary or above: 100% | Chronic illness: 9.0% | CSS | e-Health Literacy, eHL (eHEALS) | Non-significant associations between CYB and eHL |
| Mrayyan et al., 2024[72] | Jordan | Cross-sectional study | Nurses | 303 | 18-25: 72.6% | 71.3 | Tertiary or above: 89.4% | NR | CSS-SF (8 items) | Anxiety Sensitivity, AS (ASI-3)  Internet Addiction, IA (IAT) | CYB positively associates with IA (Moderator: AS) |
| Sansakorn et al., 2024[73] | Pakistan | Cross-sectional study | Adults without psychological illness | 1295 | ≥56: 19.1% | 36.3 | Tertiary or above: 54.9% | Physical disease: 47.2% | CSS-SF (12 items) | Health Anxiety, HA (SHAI)  Health Literacy, HL (HLS) | CYB positively associates with HA and HL |
| Šoštarić et al., 2024[74] | Croatia | Cross-sectional study | Pregnant women | 360 | 35.3 | 100 | Tertiary or above: 59.7% | NR | SCS | Health Anxiety, HA (SHAI)  Pregnancy-specific Anxiety, PA (PCS^2^) | CYB positively associates with HA and PA |
| Xu & Chen, 2024[75] | China | Cross-sectional study | General population (over 18 years of age) | 986 | NR | 51.7 | Tertiary or above: 94.6% | Chronic illness: 32.6% | CSS-SF (12 items) | Coping Strategy, CS (Brief-COPE)  Overuse of Health Care, OHC (Infodemic- and Misinformation-Driven Overuse of Health Care Services) | OHC → CYB (positive)  Moderator: CS  CYB negatively associates with CS |
| Amanak & Şule Bilgiç, 2025[76] | Turkey | Cross-sectional study | Pregnant women | 149 | 28.1 (4.8) | 100 | Tertiary or above: 27.5% | NR | CSS-SF (12 items) | Anxiety Sensitivity, AS (ASI-3)  Health Anxiety, HA (HAI)  Uncertainty, IU (IUS-12) | CYB positively associates with AS and HA and IU |
| Cao et al., 2025[77] | China | Cross-sectional study | General population | 531 | 25.5  [18-50] | 55.0 | Tertiary or above: 80.4% | Systemic diseases: 42.6% | CSS-SF (12 items) | Anxiety (GAD-7)  Somatic Symptom, SS (SSS-8)  Temporomandibular Disorders, TMDs (5Ts) | CYB positively associates with Anxiety, SS, and TMDs |
| Cici et al., 2025[78] | Turkey | Cross-sectional study | General population (over 18 years of age) | 158 | 31.6 (5.2) | 42.2 | Tertiary or above: 62.7% | NR | CSS-SF (12 items) | Death Anxiety, DA (DAS) | CYB positively associates with DA |
| Demir et al., 2025[79] | Turkey | Cross-sectional study | High school students | 328 | 15.6 (2.1)  [14-17] | 59.8 | Tertiary or above: 0 | NR | CSS | Health Anxiety, HA (SHAI) | CYB positively associates with HA |
| Ergün Özdel et al., 2025[80] | Turkey | Cross-sectional study | Parents of children attending the general pediatrics outpatient clinic | 619 | 25-44 yrs: 80.9% | 55.9 | Tertiary or above: 32.8% | NR | CSS | Health Literacy, HL (HLS) | CYB positively associates with HL |
| Kefeli Col et al., 2025[81] | Turkey | Cross-sectional study | University students | 568 | 20.2 (1.5) | 80.1 | Tertiary or above: 100% | Chronic illness: 3% | CSS | Death Anxiety, DA (DAS)  e-Health Literacy, eHL (eHEALS)  Health Anxiety, HA (SHAI) | CYB positively associates with DA, eHL, and HA |
| Lai et al., 2025[82] | China | Cross-sectional study | Patients living with chronic disease(s) | 827 | 42.1 (13.6)  [19-70] | 53.3 | Tertiary or above: 54.2% | Diabetes: 40.6%  Hypertension: 45.6% | CSS | e-Health Literacy, eHL (eHEALS)  Health-related social Media Use, HSMU (HINTS)  Online Patient-centered Communication, OPCC (OPCCS)  Psychological Distress, PD (PANAS) | CYB positively associates with HL, HSMU, OPCC, and PD |
| Macovei & Măirean, 2025[83] | Romania | Cross-sectional study | University students | 405 | 21.1 (3.3)  [18-38] | 67.0 | Tertiary or above: 58% | NR | CSS-SF (12 items) | Death Anxiety, DA (DAS)  Health Anxiety, HA (SHAI) | CYB positively associates with DA and HA  HA → CYB → DA |
| Xu et al., 2025[84] | China | Cross-sectional study | General population (over 18 years of age) | 2415 | 32.9 (7.7)  [18-80] | 51.4 | Tertiary or above: 82.5% | NR | CSS-SF (12 items) | Psychological Distress, PD (K-10)  Suicidal Ideation, SI (SIDAS) | CYB positively associates with PD and SI  CYB → PD → SI |
| Xu & Starcevic, 2025[85] | China | Cross-sectional study | Older Adults (60 years or older) | 638 | [60-79] | 35.9 | Tertiary or above: 48.6% | Chronic illness: 55.3% | CSS-SF (12 items) | Cognitive Fusion, CF (CFQ)  Quality of Life, QOL (EQ-5D-5L)  Mental Well-being, MW (WHO-5) | CYB negatively associates with QOL and MW, positively associates with CF  CYB → CF → QOL/MW |
| Yorulmaz et al., 2025[86] | Turkey | Cross-sectional study | General population (over 18 years of age) | 403 | 18-27 yrs: 52.9% | 69.0 | Tertiary or above: 63.8% | NR | CS | Internet Addiction, IA (IAT) | CYB positively associates with IA |
| Yurttaş et al., 2025[87] | Turkey | Cross-sectional study | Dental patients | 281 | [18-30] | 54.1 | Tertiary or above: 46.2% | NR | CSS-SF (12 items) | Dental Caries, DC (DMFT index) | Non-significant correlations between CYB and DC |

NR: No report.

ASI-3, Anxiety Sensitivity Index-3; ASQ, Anxiety Sensitivity Questionnaire; BDI, Beck Depression Inventory-II; Brief-COPE, Coping Orientation to Problems Experienced Inventory; BSI, Brief Symptom Inventory (Subscale: anxiety); BSMAS, Bergen Social Media Addiction Scale; CAS, Coronavirus Anxiety Scale; CES-D, Center for Epidemiologic Studies Depression Scale; CFQ, ; Cognitive Fusion Questionnaire; CGI, Clinical Global Improvement scale; CS, Cyberchondria Scale; CSS: Cyberchondria Severity Scale; CSS-SF, the short form of Cyberchondria Severity Scale; DAS, Death Anxiety Scale; DASS-21, Depression Anxiety Stress Scale-21; DERS, Difficulties in Emotion Regulation Scale; DMFT index, Decayed, Missing, and Filled Teeth index; DOCS, Dimensional Obsessive–Compulsive Scale; DTS, Distress Tolerance Scale; DV, Dependent Variable; ECBID, Efficacy of Coping Behaviors for Infectious Diseases Scale; ESDS, Epidemiological Studies-Depression Scale; EUROHIS-QOL-8, European Health Interview Survey QOL 8 items; FCV-19S, FAD, Family Assessment Device; Fear of COVID-19 scale; GPIUS, Generalized Problematic Internet Use Scale; HADS-A, Hospital Anxiety and Depression Scale, Anxiety subscale; HADS-D, Hospital Anxiety and Depression Scale, Depression subscale; HAI, Health Anxiety Inventory; HAQ, Health Anxiety Questionnaire; HCQ, Health Cognitions Questionnaire; HCU, Health Care Utilization questionnaire; HLS, European Health Literacy Survey Questionnaire; IAT, Internet Addiction Test; ICVVS, Intention to Get a COVID-19 Vaccination Scale; IUS-12, The Intolerance of Uncertainty Scale-short version; IV, Independent Variable; K-6, Kessler Psychological Distress Scale – 6 items; K-10, Kessler Psychological Distress Scale-10 items; LOT, The Life Orientation Test; MAC-RF, Multidimensional Assessment of COVID-19–Related Fears; MCQ-30, Metacognition Questionnaire-30; MCQ-HA, Metacognitions Questionnaire—Health Anxiety; MIHT, Multidimensional inventory of hypochondriacal traits; MOCQ, Maudsley Obsessional-Compulsive Questionnaire; MOCQ-R, Reduced Form of the Maudsley Obsessional-Compulsive Questionnaire; MSPSS, Multidimensional Scale of Perceived Social Support; OCI-R, The Obsessive-Compulsive Inventory Revised; OPCCS, Online Patient-centered Communication Scale; ORTO-11, Orthorexia Nervosa-11 scale; PANAS, Positive and Negative Affect Schedule; PCS^1^, Pain Catastrophizing Scale; PCS^2^, Pregnancy Concerns Scale; PHQ-9, The Patient Health Questionnaire – 9; PHQ-15, The Patient Health Questionnaire – 15 Somatic Symptom Severity Scale; PI, Padua Inventory; PSQI, Pittsburgh Sleep Quality Index; PSS, Perceived stress scale; PWBS, Psychological Well-Being Scale; QOLS, Quality of Life Scale; RQ, Relationship Questionnaires; RSES, The Rosenberg Self-Esteem Scale; SAS, Smartphone Addiction Scale; SCS, The short cyberchondria scale; SDS, Sheehan Disability Scale; SF-12, Quality of life short form-12 health survey; SHAI, The Short Health Anxiety Inventory; SIDAS, Suicidal Ideation Attributes Scale; SQ, Security Questionnaire; SSS-8, Somatic Symptom Scale - 8; SWS, Subjective Well-being Scale; TAS-20, The Toronto alexithymia scale; WHOQOL, WHO Quality of Life-BREF; WHO-5, World Health Organization-5 Well-Being Index; WI, Whiteley Index; WI-6, Whiteley Index-6 items; YIAT-SF, Young Internet Addiction Test-Short Form; Y-BOCS, Yale-Brown Obsessive-Compulsive Rating Scale; 5Ts, 5 Temporomandibular Disorder Symptoms

**Reference:**

1. Fergus TA: **The Cyberchondria Severity Scale (CSS): an examination of structure and relations with health anxiety in a community sample**. *J Anxiety Disord* 2014, **28**(6):504-510.

2. McElroy E, Shevlin M: **The development and initial validation of the cyberchondria severity scale (CSS)**. *J Anxiety Disord* 2014, **28**(2):259-265.

3. Fergus TA: **Anxiety sensitivity and intolerance of uncertainty as potential risk factors for cyberchondria: A replication and extension examining dimensions of each construct**. *J Affect Disord* 2015, **184**:305-309.

4. Norr AM, Albanese BJ, Oglesby ME, Allan NP, Schmidt NB: **Anxiety sensitivity and intolerance of uncertainty as potential risk factors for cyberchondria**. *J Affect Disord* 2015, **174**:64-69.

5. Norr AM, Oglesby ME, Raines AM, Macatee RJ, Allan NP, Schmidt NB: **Relationships between cyberchondria and obsessive-compulsive symptom dimensions**. *Psychiatry Res* 2015, **230**(2):441-446.

6. Barke A, Bleichhardt G, Rief W, Doering BK: **The Cyberchondria Severity Scale (CSS): German Validation and Development of a Short Form**. *Int J Behav Med* 2016, **23**(5):595-605.

7. Fergus TA, Russell LH: **Does cyberchondria overlap with health anxiety and obsessive-compulsive symptoms? An examination of latent structure and scale interrelations**. *J Anxiety Disord* 2016, **38**:88-94.

8. Fergus TA, Spada MM: **Cyberchondria: Examining relations with problematic Internet use and metacognitive beliefs**. *Clin Psychol Psychother* 2017, **24**(6):1322-1330.

9. Fergus TA, Spada MM: **Moving toward a metacognitive conceptualization of cyberchondria: Examining the contribution of metacognitive beliefs, beliefs about rituals, and stop signals**. *J Anxiety Disord* 2018, **60**:11-19.

10. Mathes BM, Norr AM, Allan NP, Albanese BJ, Schmidt NB: **Cyberchondria: Overlap with health anxiety and unique relations with impairment, quality of life, and service utilization**. *Psychiatry Res* 2018, **261**:204-211.

11. Selvi Y, Turan SG, Sayin AA, Boysan M, Kandeger A: **The Cyberchondria Severity Scale (CSS): Validity and reliability study of the Turkish version**. *Sleep and Hypnosis (Online)* 2018, **20**(4):241-246.

12. Bajcar B, Babiak J, Olchowska-Kotala A: **Cyberchondria and its measurement. The Polish adaptation and psychometric properties of the Cyberchondria Severity Scale CSS-PL**. *Psychiatr Pol* 2019, **53**(1):49-60.

13. Blackburn J, Fischerauer SF, Talaei-Khoei M, Chen NC, Oh LS, Vranceanu AM: **What are the Implications of Excessive Internet Searches for Medical Information by Orthopaedic Patients?** *Clin Orthop Relat Res* 2019, **477**(12):2665-2673.

14. Gibler RC, Jastrowski Mano KE, O'Bryan EM, Beadel JR, McLeish AC: **The role of pain catastrophizing in cyberchondria among emerging adults**. *Psychol Health Med* 2019, **24**(10):1267-1276.

15. Jokic-Begic N, Lauri Korajlija A, Mikac U: **Cyberchondria in the age of COVID-19**. *PLoS One* 2020, **15**(12):e0243704.

16. Jungmann SM, Witthöft M: **Health anxiety, cyberchondria, and coping in the current COVID-19 pandemic: Which factors are related to coronavirus anxiety?** *J Anxiety Disord* 2020, **73**:102239.

17. Maftei A, Holman AC: **Cyberchondria During the Coronavirus Pandemic: The Effects of Neuroticism and Optimism**. *Front Psychol* 2020, **11**:567345.

18. Marino C, Fergus TA, Vieno A, Bottesi G, Ghisi M, Spada MM: **Testing the Italian version of the Cyberchondria Severity Scale and a metacognitive model of cyberchondria**. *Clin Psychol Psychother* 2020, **27**(4):581-596.

19. Seyed Hashemi SG, Hosseinnezhad S, Dini S, Griffiths MD, Lin CY, Pakpour AH: **The mediating effect of the cyberchondria and anxiety sensitivity in the association between problematic internet use, metacognition beliefs, and fear of COVID-19 among Iranian online population**. *Heliyon* 2020, **6**(10):e05135.

20. Shailaja B, Shetty V, Chaudhury S, Thyloth M: **Exploring cyberchondria and its associations in dental students amid COVID-19 infodemic**. *Ind Psychiatry J* 2020, **29**(2):257-267.

21. Arsenakis S, Chatton A, Penzenstadler L, Billieux J, Berle D, Starcevic V, Viswasam K, Khazaal Y: **Unveiling the relationships between cyberchondria and psychopathological symptoms**. *J Psychiatr Res* 2021, **143**:254-261.

22. Bajcar B, Babiak J: **Self-esteem and cyberchondria: The mediation effects of health anxiety and obsessive–compulsive symptoms in a community sample**. *Current Psychology* 2021, **40**(6):2820-2831.

23. Durak Batıgün A, Şenkal Ertürk İ, Gör N, Kömürcü Akik B: **The pathways from distress tolerance to Cyberchondria: A multiple-group path model of young and middle adulthood samples**. *Curr Psychol* 2021, **40**(11):5718-5726.

24. Han L, Zhan Y, Li W, Xu Y, Xu Y, Zhao J: **Associations Between the Perceived Severity of the COVID-19 Pandemic, Cyberchondria, Depression, Anxiety, Stress, and Lockdown Experience: Cross-sectional Survey Study**. *JMIR Public Health Surveill* 2021, **7**(9):e31052.

25. Oniszczenko W: **Anxious temperament and cyberchondria as mediated by fear of COVID-19 infection: A cross-sectional study**. *PLoS One* 2021, **16**(8):e0255750.

26. Peng XQ, Chen Y, Zhang YC, Liu F, He HY, Luo T, Dai PP, Xie WZ, Luo AJ: **The Status and Influencing Factors of Cyberchondria During the COVID-19 Epidemic. A Cross-Sectional Study in Nanyang City of China**. *Front Psychol* 2021, **12**:712703.

27. Rahme C, Akel M, Obeid S, Hallit S: **Cyberchondria severity and quality of life among Lebanese adults: the mediating role of fear of COVID-19, depression, anxiety, stress and obsessive-compulsive behavior-a structural equation model approach**. *BMC Psychol* 2021, **9**(1):169.

28. Sarıgedik E, Ölmez SB: **The investigation of the relationships among coronavirus anxiety, cyberchondria, and online shopping**. *Konuralp Medical Journal* 2021, **13**(S1):446-454.

29. Vismara M, Vitella D, Biolcati R, Ambrosini F, Pirola V, Dell'Osso B, Truzoli R: **The Impact of COVID-19 Pandemic on Searching for Health-Related Information and Cyberchondria on the General Population in Italy**. *Front Psychiatry* 2021, **12**:754870.

30. Wu X, Nazari N, Griffiths MD: **Using Fear and Anxiety Related to COVID-19 to Predict Cyberchondria: Cross-sectional Survey Study**. *J Med Internet Res* 2021, **23**(6):e26285.

31. Abu Khait A, Mrayyan MT, Al-Rjoub S, Rababa M, Al-Rawashdeh S: **Cyberchondria, Anxiety Sensitivity, Hypochondria, and Internet Addiction: Implications for Mental Health Professionals**. *Curr Psychol* 2022:1-12.

32. Afrin R, Prybutok G: **Insights into the antecedents of cyberchondria: a perspective from the USA**. *Health Promot Int* 2022, **37**(4).

33. Ahorsu DK, Lin CY, Alimoradi Z, Griffiths MD, Chen HP, Broström A, Timpka T, Pakpour AH: **Cyberchondria, Fear of COVID-19, and Risk Perception Mediate the Association between Problematic Social Media Use and Intention to Get a COVID-19 Vaccine**. *Vaccines (Basel)* 2022, **10**(1).

34. Airoldi S, Kolubinski DC, Nikčević AV, Spada MM: **The relative contribution of health cognitions and metacognitions about health anxiety to cyberchondria: A prospective study**. *J Clin Psychol* 2022, **78**(5):809-820.

35. Ambrosini F, Truzoli R, Vismara M, Vitella D, Biolcati R: **The effect of cyberchondria on anxiety, depression and quality of life during COVID-19: the mediational role of obsessive-compulsive symptoms and Internet addiction**. *Heliyon* 2022, **8**(5):e09437.

36. Błachnio A, Przepiórka A, Kot P, Cudo A, Steuden S: **The role of emotional functioning in the relationship between health anxiety and cyberchondria**. *Curr Psychol* 2022:1-11.

37. Bottesi G, Marino C, Vieno A, Ghisi M, Spada MM: **Psychological distress in the context of the COVID-19 pandemic: the joint contribution of intolerance of uncertainty and cyberchondria**. *Psychol Health* 2022, **37**(11):1396-1413.

38. Boysan M, Eşkisu M, Çam Z: **Relationships between fear of COVID-19, cyberchondria, intolerance of uncertainty, and obsessional probabilistic inferences: A structural equation model**. *Scand J Psychol* 2022, **63**(5):439-448.

39. Ciułkowicz M, Misiak B, Szcześniak D, Grzebieluch J, Maciaszek J, Rymaszewska J: **Social Support Mediates the Association between Health Anxiety and Quality of Life: Findings from a Cross-Sectional Study**. *Int J Environ Res Public Health* 2022, **19**(19).

40. Durmuş A, Deniz S, Akbolat M, Çimen M: **Does Cyberchondria Mediate the Effect of COVID-19 Fear on the Stress?** *Soc Work Public Health* 2022, **37**(4):356-369.

41. Karakaş N, Tekin Ç, Bentli R, Demir E: **Cyberchondria, Covid-19 phobia, and well-being: a relational study on teachers**. *Med Lav* 2022, **113**(3):e2022027.

42. Liu S, Yang H, Cheng M, Miao T: **Family Dysfunction and Cyberchondria among Chinese Adolescents: A Moderated Mediation Model**. *Int J Environ Res Public Health* 2022, **19**(15).

43. Nadeem F, Malik NI, Atta M, Ullah I, Martinotti G, Pettorruso M, Vellante F, Di Giannantonio M, De Berardis D: **Relationship between Health-Anxiety and Cyberchondria: Role of Metacognitive Beliefs**. *J Clin Med* 2022, **11**(9).

44. Rashid Z, Rathore MA, Khushk IA, Mashhadi SF, Ahmed M, Shahzeb M: **Intolerance of Uncertainty and Anxiety Sensitivity as Prospective Risk Factors for Cyberchondria in Undergraduate Students**. *Annals of King Edward Medical University* 2022, **28**(1):91-96.

45. Santoro G, Starcevic V, Scalone A, Cavallo J, Musetti A, Schimmenti A: **The doctor is in (ternet): the mediating role of health anxiety in the relationship between somatic symptoms and cyberchondria**. *Journal of personalized medicine* 2022, **12**(9):1490.

46. Sezer Ö, Başoğlu MA, Dağdeviren HN: **An examination of cyberchondria’s relationship with trait anxiety and psychological well-being in women of reproductive age: A cross-sectional study**. *Medicine* 2022, **101**(46):e31503.

47. Vismara M, Benatti B, Ferrara L, Colombo A, Bosi M, Varinelli A, Pellegrini L, Viganò C, Fineberg NA, Dell’Osso B: **A preliminary investigation of cyberchondria and its correlates in a clinical sample of patients with obsessive–compulsive disorder, anxiety and depressive disorders attending a tertiary psychiatric clinic**. *International Journal of Psychiatry in Clinical Practice* 2022, **26**(2):111-122.

48. Yalçın İ, Boysan M, Eşkisu M, Çam Z: **Health anxiety model of cyberchondria, fears, obsessions, sleep quality, and negative affect during COVID-19**. *Curr Psychol* 2022:1-18.

49. Zhou Y, Dai L, Deng Y, Zeng H, Yang L: **The moderating effect of alexithymia on the relationship between stress and cyberchondria**. *Front Psychiatry* 2022, **13**:1043521.

50. Zolotareva A: **Cyberchondria, but not preventive behavior, mediates the relationship between fear of COVID-19 and somatic burden: Evidence from Russia**. *Front Psychiatry* 2022, **13**:1018659.

51. Błachnio A, Przepiórka A, Kot P, Cudo A, McElroy E: **The mediating role of rumination between stress appraisal and cyberchondria**. *Acta Psychologica* 2023, **238**:103946.

52. El-Zayat A, Namnkani SA, Alshareef NA, Mustfa MM, Eminaga NS, Algarni GA: **Cyberchondria and its Association with Smartphone Addiction and Electronic Health Literacy among a Saudi Population**. *Saudi J Med Med Sci* 2023, **11**(2):162-168.

53. Eşkisu M, Çam Z, Boysan M: **Health-Related Cognitions and Metacognitions Indirectly Contribute to the Relationships Between Impulsivity, Fear of COVID-19, and Cyberchondria**. *J Ration Emot Cogn Behav Ther* 2023:1-23.

54. Infanti A, Starcevic V, Schimmenti A, Khazaal Y, Karila L, Giardina A, Flayelle M, Razavi SBH, Baggio S, Vögele C: **Predictors of cyberchondria during the COVID-19 pandemic: cross-sectional study using supervised machine learning**. *JMIR formative research* 2023, **7**(1):e42206.

55. Jeong GC, Lee K, Jin Y: **Effects of the Fear of COVID-19 and Efficacy of Coping Behavior for Infectious Diseases after the End of COVID-19: Moderating Effects of Cyberchondria and eHealth Literacy**. *Behav Sci (Basel)* 2023, **13**(8).

56. Liu Y, Peng W, Cao M, Zhang S, Peng J, Zhou Z: **Cyberchondria and Chinese Adolescent Mental Health in the Age of COVID-19 Pandemic**. *Cyberpsychol Behav Soc Netw* 2023, **26**(8):631-639.

57. Nasiri M, Mohammadkhani S, Akbari M, Alilou MM: **The structural model of cyberchondria based on personality traits, health-related metacognition, cognitive bias, and emotion dysregulation**. *Frontiers in Psychiatry* 2023, **13**:960055.

58. Tarabay C, Bitar Z, Akel M, Hallit S, Obeid S, Soufia M: **Cyberchondria Severity and Quality of Life Among Lebanese Adults: The Moderating Effect of Emotions**. *Prim Care Companion CNS Disord* 2023, **25**(2).

59. Varer Akpinar C, Mandiracioglu A, Ozvurmaz S, Kurt F, Koc N: **Cyberchondria and COVID-19 anxiety and internet addiction among nursing students**. *Current Psychology* 2023, **42**(3):2406-2414.

60. Vujić A, Volarov M, Latas M, Demetrovics Z, Kiraly O, Szabo A: **Are Cyberchondria and Intolerance of Uncertainty Related to Smartphone Addiction?** *Int J Ment Health Addict* 2023:1-19.

61. Wang D, Sun L, Shao Y, Zhang X, Maguire P, Hu Y: **Research and Evaluation of a Cyberchondria Severity Scale in a Chinese Context**. *Psychol Res Behav Manag* 2023, **16**:4417-4429.

62. Yam FC, Korkmaz O, Griffiths MD: **The association between fear of Covid-19 and smartphone addiction among individuals: the mediating and moderating role of cyberchondria severity**. *Curr Psychol* 2023, **42**(3):2377-2390.

63. Zhu X, Zheng T, Ding L, Zhang X: **Exploring associations between eHealth literacy, cyberchondria, online health information seeking and sleep quality among university students: A cross-section study**. *Heliyon* 2023, **9**(6):e17521.

64. Agrawal V, Khulbe Y, Singh A, Kar SK: **The digital health dilemma: Exploring cyberchondria, well-being, and smartphone addiction in medical and non-medical undergraduates**. *Indian J Psychiatry* 2024, **66**(3):256-262.

65. Ali SS, Hendawi NE, El-Ashry AM, Mohammed MS: **The relationship between cyberchondria and health literacy among first-year nursing students: the mediating effect of health anxiety**. *BMC Nurs* 2024, **23**(1):776.

66. Atsizata M, Sögüt SC: **The relationship between orthorexia nervosa and cyberchondria levels in nurses: A cross-sectional study**. *Arch Psychiatr Nurs* 2024, **48**:30-35.

67. El-Zoghby SM, Zaghloul NM, Tawfik AM, Elsherbiny NM, Shehata SA, Soltan EM: **Cyberchondria and smartphone addiction: A correlation survey among undergraduate medical students in Egypt**. *J Egypt Public Health Assoc* 2024, **99**(1):7.

68. Fang J, Qiu C, Sun Z, Zhou J, He P, Conti A, Lu Y, Huang X, Xu J, Tang W: **A national survey of pandemic fear and cyberchondria after ending zero-COVID policy: The chain mediating role of alexithymia and psychological distress**. *Compr Psychiatry* 2024, **133**:152505.

69. Fang S, Mushtaque I: **The Moderating Role of Health Literacy and Health Promoting Behavior in the Relationship Among Health Anxiety, Emotional Regulation, and Cyberchondria**. *Psychology Research and Behavior Management* 2024:51-62.

70. Jungmann SM, Gropalis M, Schenkel SK, Witthöft M: **Is cyberchondria specific to hypochondriasis?** *Journal of Anxiety Disorders* 2024, **102**:102798.

71. Kalantari A, Valizadeh-Haghi S, Starcevic V, Shahbodaghi A, Rahmatizadeh S, Zayeri F, Khazaal Y: **The relationship between e-Health literacy and cyberchondria in Iranian students of health sciences**. *Front Psychiatry* 2024, **15**:1421391.

72. Mrayyan MT, Abu Khait A, Al-Mrayat Y, Alkhawaldeh JfM, Alfayoumi I, Algunmeeyn A, Kutah OA, Abunab HY, Hamdan MS, Alhabashneh H: **Anxiety sensitivity moderates the relationship between internet addiction and cyberchondria among nurses**. *Journal of Health Psychology* 2024:13591053241249634.

73. Sansakorn P, Mushtaque I, Awais EYM, Dost MKB: **The Relationship between Cyberchondria and Health Anxiety and the Moderating Role of Health Literacy among the Pakistani Public**. *Int J Environ Res Public Health* 2024, **21**(9).

74. Šoštarić M, Jokić-Begić N, Vukušić Mijačika M: **Can't stop, won't stop - understanding anxiety's role in cyberchondria among pregnant women**. *Women Health* 2024, **64**(2):185-194.

75. Xu RH, Chen C: **Moderating Effect of Coping Strategies on the Association Between the Infodemic-Driven Overuse of Health Care Services and Cyberchondria and Anxiety: Partial Least Squares Structural Equation Modeling Study**. *Journal of Medical Internet Research* 2024, **26**:e53417.

76. Amanak K, Şule Bilgiç F: **Cyberchondria and pregnancy-related anxiety: multidimensional assessment of Health anxiety, sensitivity, uncertainty, and fear of childbirth in pregnant women**. *Psychol Health Med* 2025:1-15.

77. Cao Z, Sun Y, Li H, Lin C, Wong HL, Ming H, Wo PK, Wang J, Xiong X: **From Cyberchondria to Temporomandibular Disorders: How Somatic Symptoms and Anxiety Bridge the Gap**. *J Oral Rehabil* 2025, **52**(10):1777-1787.

78. Cіcі R, Topdemіr EA, Kapikiran G: **The Relationship Between Preoperative Cyberchondria Levels and Death Anxiety of Liver Donors**. *Nurs Health Sci* 2025, **27**(3):e70179.

79. Demir G, Bahar Z, Yildirim D: **Evaluation of health anxiety and cyberchondria levels in adolescent high school students**. *J Child Adolesc Ment Health* 2025:1-17.

80. Ergün Özdel ZG, Özkaya G, Türe Ş: **Parental health information seeking online: How cyberchondria and health literacy shape pediatric health decisions**. *Arch Argent Pediatr* 2025:e202510771.

81. Kefeli Col B, Gumusler Basaran A, Genc Kose B: **The Relationship Between E-Health Literacy, Health Anxiety, Cyberchondria, and Death Anxiety in University Students That Study in Health Related Department**. *J Multidiscip Healthc* 2025, **18**:1581-1595.

82. Lai YK, Lai Z, Zhao X: **Counteracting cyberchondria in Chinese chronic disease patients: The divergent roles of health-related social media use and online patient-centered communication**. *Patient Educ Couns* 2025, **141**:109337.

83. Macovei M, Măirean C: **Health anxiety and death anxiety: The role of cyberchondria and social aspirations**. *J Health Psychol* 2025:13591053251341191.

84. Xu RH, Liang X, Starcevic V: **Exploring the Relationship Between Cyberchondria and Suicidal Ideation: Cross-Sectional Mediation Analysis**. *J Med Internet Res* 2025, **27**:e72414.

85. Xu RH, Starcevic V: **Cyberchondria in Older Adults and Its Relationship With Cognitive Fusion, Health-Related Quality of Life, and Mental Well-Being: Mediation Analysis**. *JMIR Aging* 2025, **8**:e70302.

86. Yorulmaz M, Göde A, Aydoğdu A, Dilekçi R: **Investigation of the effect of internet addiction on cyberchondria**. *Psychol Health Med* 2025, **30**(6):1187-1198.

87. Yurttaş M, İzgi E, Gürbüz E: **Analysis of relationship between cyberchondria levels and oral health in dental patients**. *BMC Oral Health* 2025, **25**(1):1476.
