# Supplementary material for: Impact of Cyberchondria on Health and Quality of Life: Scoping Review
Source: J Med Internet Res. 2025 Dec 4;27:e77977. doi: 10.2196/77977 (PMC12715475; doi:10.2196/77977)
Supplement: Multimedia Appendix 4 [file jmir_v27i1e77977_app4.docx]

**Appendix 3. Risk of bias assessment results**

|  | **Study sample selection (max. 2)** | | **Assessment of exposure and outcome (max. 4)** | | **Confounding factor (max. 3)** | | **Overall rating** |
| --- | --- | --- | --- | --- | --- | --- | --- |
|  | Representativeness | Sample size | Assessment of exposure | Assessment of outcome | Adjustment for confounder(s) | Assessment for confounder(s) |  |
| Fergus, 2014 [1] | 1 | 1 | 1 | 1 | 2 | 1 | 7 |
| McElroy & Shevlin, 2014 [2] | 1 | 1 | 1 | 1 | 0 | 0 | 4 |
| Fergus, 2015 [3] | 1 | 1 | 1 | 1 | 0 | 0 | 4 |
| Norr et al., 2015a [4] | 1 | 1 | 1 | 1 | 1 | 1 | 6 |
| Norr et al., 2015b [5] | 1 | 1 | 1 | 1 | 1 | 1 | 6 |
| Barke et al., 2016 [6] | 0 | 1 | 1 | 1 | 0 | 0 | 3 |
| Fergus & Russell, 2016 [7] | 1 | 1 | 1 | 1 | 2 | 1 | 7 |
| Fergus & Spada, 2017 [8] | 1 | 1 | 1 | 1 | 2 | 1 | 7 |
| Fergus & Spada, 2018a [9] | 0 | 1 | 1 | 1 | 0 | 0 | 3 |
| Fergus & Spada, 2018b [9] | 1 | 1 | 1 | 1 | 0 | 0 | 4 |
| Mathes et al., 2018 [10] | 1 | 1 | 1 | 1 | 1 | 1 | 6 |
| Selvi et al., 2018 [11] | 0 | 1 | 1 | 1 | 0 | 0 | 3 |
| Bajcar et al., 2019 [12] | 1 | 1 | 1 | 1 | 0 | 0 | 4 |
| Blackburn et al., 2019 [13] | 1 | 1 | 1 | 1 | 1 | 1 | 6 |
| Gibler et al., 2019 [14] | 0 | 1 | 1 | 1 | 2 | 1 | 6 |
| Jokic-Begic et al., 2020 [15] | 1 | 1 | 1 | 1 | 0 | 0 | 4 |
| Jungmann & Witthöft, 2020 [16] | 1 | 1 | 1 | 1 | 2 | 1 | 7 |
| Maftei & Holman, 2020 [17] | 1 | 1 | 1 | 1 | 1 | 1 | 6 |
| Marino et al., 2020 [18] | 1 | 1 | 1 | 1 | 2 | 1 | 7 |
| Seyed et al., 2020 [19] | 1 | 1 | 1 | 1 | 1 | 1 | 6 |
| Shailaja et al., 2020 [20] | 1 | 1 | 1 | 1 | 2 | 1 | 7 |
| Arsenakis et al., 2021 [21] | 1 | 1 | 1 | 1 | 2 | 1 | 7 |
| Bajcar & Babiak, 2021 [22] | 1 | 1 | 1 | 1 | 1 | 1 | 6 |
| Durak Batıgün et al., 2021 [23] | 1 | 1 | 1 | 1 | 0 | 0 | 4 |
| Han et al., 2021 [24] | 1 | 1 | 1 | 1 | 1 | 1 | 6 |
| Oniszczenko, 2021 [25] | 1 | 1 | 1 | 1 | 0 | 0 | 4 |
| Peng et al., 2021 [26] | 1 | 1 | 1 | 1 | 2 | 1 | 7 |
| Rahme et al., 2021 [27] | 1 | 1 | 1 | 1 | 1 | 1 | 6 |
| Sarigedik & Olmez, 2021 [28] | 1 | 1 | 1 | 1 | 0 | 0 | 4 |
| Vismara et al., 2021 [29] | 1 | 1 | 1 | 1 | 2 | 1 | 7 |
| Wu et al., 2021 [30] | 1 | 1 | 1 | 1 | 2 | 1 | 7 |
| Abu Khait et al., 2022 [31] | 0 | 1 | 1 | 1 | 1 | 1 | 5 |
| Afrin & Prybutok, 2022 [32] | 1 | 1 | 1 | 1 | 1 | 1 | 6 |
| Ahorsu et al., 2022 [33] | 1 | 1 | 1 | 1 | 2 | 1 | 7 |
| Airoldi et al., 2022 [34] | 1 | 1 | 1 | 1 | 1 | 1 | 6 |
| Ambrosini et al., 2022 [35] | 1 | 1 | 1 | 1 | 1 | 1 | 6 |
| Błachnio et al., 2022 [36] | 1 | 1 | 1 | 1 | 1 | 1 | 6 |
| Bottesi et al., 2022a [37] | 1 | 1 | 1 | 1 | 1 | 1 | 6 |
| Bottesi et al., 2022b [37] | 1 | 1 | 1 | 1 | 1 | 1 | 6 |
| Boysan et al., 2022 [38] | 1 | 1 | 1 | 1 | 1 | 1 | 6 |
| Ciułkowicz et al., 2022 [39] | 1 | 1 | 1 | 1 | 2 | 1 | 7 |
| Durmuş et al., 2022 [40] | 1 | 1 | 1 | 1 | 1 | 1 | 6 |
| Karakaş et al., 2022 [41] | 1 | 1 | 1 | 1 | 1 | 1 | 6 |
| Liu et al., 2022 [42] | 1 | 1 | 1 | 1 | 2 | 1 | 7 |
| Nadeem et al., 2022 [43] | 0 | 1 | 1 | 1 | 0 | 0 | 3 |
| Rashid et al., 2022 [44] | 1 | 1 | 1 | 1 | 1 | 1 | 6 |
| Santoro et al., 2022 [45] | 1 | 1 | 1 | 1 | 1 | 1 | 6 |
| Sezer et al., 2022 [46] | 1 | 1 | 1 | 1 | 2 | 1 | 7 |
| Vismara et al., 2022 [47] | 1 | 1 | 1 | 1 | 1 | 1 | 6 |
| Yalçın et al., 2022 [48] | 1 | 1 | 1 | 1 | 1 | 1 | 6 |
| Zhou et al., 2022 [49] | 1 | 1 | 1 | 1 | 1 | 1 | 6 |
| Zolotareva, 2022 [50] | 1 | 1 | 1 | 1 | 0 | 0 | 4 |
| Błachnio et al., 2023 [51] | 1 | 1 | 1 | 1 | 1 | 1 | 6 |
| El-Zayat et al., 2023 [52] | 1 | 1 | 1 | 1 | 2 | 1 | 7 |
| Eşkisu et al., 2023 [53] | 1 | 1 | 1 | 1 | 2 | 1 | 7 |
| Infanti et al., 2023 [54] | 1 | 1 | 1 | 1 | 2 | 1 | 7 |
| Jeong et al., 2023 [55] | 1 | 1 | 1 | 1 | 2 | 1 | 7 |
| Liu et al., 2023 [56] | 1 | 1 | 1 | 1 | 2 | 1 | 7 |
| Nasiri et al., 2023 [57] | 1 | 1 | 1 | 1 | 1 | 1 | 6 |
| Tarabay et al., 2023 [58] | 1 | 1 | 1 | 1 | 1 | 1 | 6 |
| Varer Akpinar et al., 2023 [59] | 1 | 1 | 1 | 1 | 1 | 1 | 6 |
| Vujić et al., 2023 [60] | 1 | 1 | 1 | 1 | 2 | 1 | 7 |
| Wang et al., 2023a [61] | 0 | 1 | 1 | 1 | 0 | 0 | 3 |
| Wang et al., 2023b [61] | 0 | 1 | 1 | 1 | 0 | 0 | 3 |
| Yam et al., 2023 [62] | 1 | 1 | 1 | 1 | 0 | 0 | 4 |
| Zhu et al., 2023 [63] | 1 | 1 | 1 | 1 | 2 | 1 | 7 |
| Agrawal et al., 2024 [64] | 1 | 1 | 1 | 1 | 0 | 0 | 4 |
| Ali et al., 2024 [65] | 1 | 1 | 1 | 1 | 2 | 1 | 7 |
| Atsizata & Sögüt, 2024 [66] | 1 | 1 | 1 | 1 | 1 | 1 | 6 |
| El-Zoghby et al., 2024 [67] | 1 | 1 | 1 | 1 | 2 | 1 | 7 |
| Fang et al., 2024 [68] | 1 | 1 | 1 | 1 | 2 | 1 | 7 |
| Fang & Mushtaque, 2024 [69] | 1 | 1 | 1 | 1 | 2 | 1 | 7 |
| Jungmann et al., 2024 [70] | 1 | 1 | 1 | 1 | 2 | 1 | 7 |
| Kalantari et al., 2024 [71] | 0 | 1 | 1 | 1 | 0 | 0 | 3 |
| Mrayyan et al., 2024 [72] | 1 | 1 | 1 | 1 | 2 | 1 | 7 |
| Sansakorn et al., 2024 [73] | 1 | 1 | 1 | 1 | 1 | 1 | 6 |
| Šoštarić et al., 2024 [74] | 1 | 1 | 1 | 1 | 2 | 1 | 7 |
| Xu & Chen, 2024 [75] | 1 | 1 | 1 | 1 | 1 | 1 | 6 |
| Amanak & Şule Bilgiç, 2025 [76] | 1 | 1 | 1 | 1 | 0 | 0 | 4 |
| Cao et al., 2025 [77] | 1 | 1 | 1 | 1 | 2 | 1 | 7 |
| Cici et al., 2025 [78] | 1 | 1 | 1 | 1 | 0 | 0 | 4 |
| Demir et al., 2025 [79] | 1 | 1 | 1 | 1 | 2 | 1 | 7 |
| Ergün Özdel et al., 2025 [80] | 0 | 1 | 1 | 1 | 0 | 0 | 3 |
| Kefeli Col et al., 2025 [81] | 1 | 1 | 1 | 1 | 0 | 0 | 4 |
| Lai et al., 2025 [82] | 1 | 1 | 1 | 1 | 2 | 1 | 7 |
| Macovei & Măirean, 2025 [83] | 0 | 1 | 1 | 1 | 1 | 1 | 5 |
| Xu et al., 2025 [84] | 1 | 1 | 1 | 1 | 2 | 1 | 7 |
| Xu & Starcevic, 2025 [85] | 1 | 1 | 1 | 1 | 2 | 1 | 7 |
| Yorulmaz et al., 2025 [86] | 1 | 1 | 1 | 1 | 0 | 0 | 4 |
| Yurttaş et al., 2025 [87] | 1 | 1 | 1 | 1 | 0 | 0 | 4 |

**Reference**

1. Fergus TA: **The Cyberchondria Severity Scale (CSS): an examination of structure and relations with health anxiety in a community sample**. *Journal of anxiety disorders* 2014, **28**(6):504-510.

2. McElroy E, Shevlin M: **The development and initial validation of the cyberchondria severity scale (CSS)**. *J Anxiety Disord* 2014, **28**(2):259-265.

3. Fergus TA: **Anxiety sensitivity and intolerance of uncertainty as potential risk factors for cyberchondria: A replication and extension examining dimensions of each construct**. *Journal of affective disorders* 2015, **184**:305-309.

4. Norr AM, Albanese BJ, Oglesby ME, Allan NP, Schmidt NB: **Anxiety sensitivity and intolerance of uncertainty as potential risk factors for cyberchondria**. *J Affect Disord* 2015, **174**:64-69.

5. Norr AM, Oglesby ME, Raines AM, Macatee RJ, Allan NP, Schmidt NB: **Relationships between cyberchondria and obsessive-compulsive symptom dimensions**. *Psychiatry research* 2015, **230**(2):441-446.

6. Barke A, Bleichhardt G, Rief W, Doering BK: **The Cyberchondria Severity Scale (CSS): German Validation and Development of a Short Form**. *Int J Behav Med* 2016, **23**(5):595-605.

7. Fergus TA, Russell LH: **Does cyberchondria overlap with health anxiety and obsessive–compulsive symptoms? An examination of latent structure and scale interrelations**. *Journal of anxiety disorders* 2016, **38**:88-94.

8. Fergus TA, Spada MM: **Cyberchondria: Examining relations with problematic Internet use and metacognitive beliefs**. *Clinical psychology & psychotherapy* 2017, **24**(6):1322-1330.

9. Fergus TA, Spada MM: **Moving toward a metacognitive conceptualization of cyberchondria: Examining the contribution of metacognitive beliefs, beliefs about rituals, and stop signals**. *J Anxiety Disord* 2018, **60**:11-19.

10. Mathes BM, Norr AM, Allan NP, Albanese BJ, Schmidt NB: **Cyberchondria: Overlap with health anxiety and unique relations with impairment, quality of life, and service utilization**. *Psychiatry research* 2018, **261**:204-211.

11. Selvi Y, Turan SG, Sayin AA, Boysan M, Kandeger A: **The Cyberchondria Severity Scale (CSS): Validity and reliability study of the Turkish version**. *Sleep and Hypnosis (Online)* 2018, **20**(4):241-246.

12. Bajcar B, Babiak J, Olchowska-Kotala A: **Cyberchondria and its measurement. The Polish adaptation and psychometric properties of the Cyberchondria Severity Scale CSS-PL**. *Psychiatr Pol* 2019, **53**(1):49-60.

13. Blackburn J, Fischerauer SF, Talaei-Khoei M, Chen NC, Oh LS, Vranceanu A-M: **What are the implications of excessive internet searches for medical information by orthopaedic patients?** *Clinical Orthopaedics and Related Research®* 2019, **477**(12):2665-2673.

14. Gibler RC, Jastrowski Mano KE, O'Bryan EM, Beadel JR, McLeish AC: **The role of pain catastrophizing in cyberchondria among emerging adults**. *Psychol Health Med* 2019, **24**(10):1267-1276.

15. Jokic-Begic N, Lauri Korajlija A, Mikac U: **Cyberchondria in the age of COVID-19**. *PLoS One* 2020, **15**(12):e0243704.

16. Jungmann SM, Witthöft M: **Health anxiety, cyberchondria, and coping in the current COVID-19 pandemic: Which factors are related to coronavirus anxiety?** *Journal of anxiety disorders* 2020, **73**:102239.

17. Maftei A, Holman AC: **Cyberchondria During the Coronavirus Pandemic: The Effects of Neuroticism and Optimism**. *Front Psychol* 2020, **11**:567345.

18. Marino C, Fergus TA, Vieno A, Bottesi G, Ghisi M, Spada MM: **Testing the Italian version of the Cyberchondria Severity Scale and a metacognitive model of cyberchondria**. *Clin Psychol Psychother* 2020, **27**(4):581-596.

19. Seyed Hashemi SG, Hosseinnezhad S, Dini S, Griffiths MD, Lin CY, Pakpour AH: **The mediating effect of the cyberchondria and anxiety sensitivity in the association between problematic internet use, metacognition beliefs, and fear of COVID-19 among Iranian online population**. *Heliyon* 2020, **6**(10):e05135.

20. Shailaja B, Shetty V, Chaudhury S, Thyloth M: **Exploring cyberchondria and its associations in dental students amid COVID-19 infodemic**. *Ind Psychiatry J* 2020, **29**(2):257-267.

21. Arsenakis S, Chatton A, Penzenstadler L, Billieux J, Berle D, Starcevic V, Viswasam K, Khazaal Y: **Unveiling the relationships between cyberchondria and psychopathological symptoms**. *Journal of Psychiatric Research* 2021, **143**:254-261.

22. Bajcar B, Babiak J: **Self-esteem and cyberchondria: The mediation effects of health anxiety and obsessive–compulsive symptoms in a community sample**. *Current Psychology* 2021, **40**(6):2820-2831.

23. Durak Batıgün A, Şenkal Ertürk İ, Gör N, Kömürcü Akik B: **The pathways from distress tolerance to Cyberchondria: A multiple-group path model of young and middle adulthood samples**. *Curr Psychol* 2021, **40**(11):5718-5726.

24. Han L, Zhan Y, Li W, Xu Y, Xu Y, Zhao J: **Associations between the perceived severity of the COVID-19 pandemic, cyberchondria, depression, anxiety, stress, and lockdown experience: cross-sectional survey study**. *JMIR public health and surveillance* 2021, **7**(9):e31052.

25. Oniszczenko W: **Anxious temperament and cyberchondria as mediated by fear of COVID-19 infection: A cross-sectional study**. *PLoS One* 2021, **16**(8):e0255750.

26. Peng XQ, Chen Y, Zhang YC, Liu F, He HY, Luo T, Dai PP, Xie WZ, Luo AJ: **The Status and Influencing Factors of Cyberchondria During the COVID-19 Epidemic. A Cross-Sectional Study in Nanyang City of China**. *Front Psychol* 2021, **12**:712703.

27. Rahme C, Akel M, Obeid S, Hallit S: **Cyberchondria severity and quality of life among Lebanese adults: the mediating role of fear of COVID-19, depression, anxiety, stress and obsessive–compulsive behavior—a structural equation model approach**. *BMC psychology* 2021, **9**:1-12.

28. Sarıgedik E, Ölmez SB: **The investigation of the relationships among coronavirus anxiety, cyberchondria, and online shopping**. *Konuralp Medical Journal* 2021, **13**(S1):446-454.

29. Vismara M, Vitella D, Biolcati R, Ambrosini F, Pirola V, Dell'Osso B, Truzoli R: **The impact of COVID-19 pandemic on searching for health-related information and cyberchondria on the general population in Italy**. *Frontiers in Psychiatry* 2021, **12**:754870.

30. Wu X, Nazari N, Griffiths MD: **Using fear and anxiety related to COVID-19 to predict cyberchondria: Cross-sectional survey study**. *Journal of Medical Internet Research* 2021, **23**(6):e26285.

31. Abu Khait A, Mrayyan MT, Al-Rjoub S, Rababa M, Al-Rawashdeh S: **Cyberchondria, Anxiety Sensitivity, Hypochondria, and Internet Addiction: Implications for Mental Health Professionals**. *Curr Psychol* 2022:1-12.

32. Afrin R, Prybutok G: **Insights into the antecedents of cyberchondria: a perspective from the USA**. *Health promotion international* 2022, **37**(4):daac108.

33. Ahorsu DK, Lin CY, Alimoradi Z, Griffiths MD, Chen HP, Broström A, Timpka T, Pakpour AH: **Cyberchondria, Fear of COVID-19, and Risk Perception Mediate the Association between Problematic Social Media Use and Intention to Get a COVID-19 Vaccine**. *Vaccines (Basel)* 2022, **10**(1).

34. Airoldi S, Kolubinski DC, Nikčević AV, Spada MM: **The relative contribution of health cognitions and metacognitions about health anxiety to cyberchondria: A prospective study**. *J Clin Psychol* 2022, **78**(5):809-820.

35. Ambrosini F, Truzoli R, Vismara M, Vitella D, Biolcati R: **The effect of cyberchondria on anxiety, depression and quality of life during COVID-19: the mediational role of obsessive-compulsive symptoms and Internet addiction**. *Heliyon* 2022, **8**(5).

36. Błachnio A, Przepiórka A, Kot P, Cudo A, Steuden S: **The role of emotional functioning in the relationship between health anxiety and cyberchondria**. *Curr Psychol* 2022:1-11.

37. Bottesi G, Marino C, Vieno A, Ghisi M, Spada MM: **Psychological distress in the context of the COVID-19 pandemic: the joint contribution of intolerance of uncertainty and cyberchondria**. *Psychology & health* 2022, **37**(11):1396-1413.

38. Boysan M, Eşkisu M, Çam Z: **Relationships between fear of COVID-19, cyberchondria, intolerance of uncertainty, and obsessional probabilistic inferences: A structural equation model**. *Scand J Psychol* 2022, **63**(5):439-448.

39. Ciułkowicz M, Misiak B, Szcześniak D, Grzebieluch J, Maciaszek J, Rymaszewska J: **Social Support Mediates the Association between Health Anxiety and Quality of Life: Findings from a Cross-Sectional Study**. *Int J Environ Res Public Health* 2022, **19**(19).

40. Durmuş A, Deniz S, Akbolat M, Çimen M: **Does Cyberchondria Mediate the Effect of COVID-19 Fear on the Stress?** *Soc Work Public Health* 2022, **37**(4):356-369.

41. Karakaş N, Tekin Ç, Bentli R, Demir E: **Cyberchondria, Covid-19 phobia, and well-being: a relational study on teachers**. *Med Lav* 2022, **113**(3):e2022027.

42. Liu S, Yang H, Cheng M, Miao T: **Family Dysfunction and Cyberchondria among Chinese Adolescents: A Moderated Mediation Model**. *Int J Environ Res Public Health* 2022, **19**(15).

43. Nadeem F, Malik NI, Atta M, Ullah I, Martinotti G, Pettorruso M, Vellante F, Di Giannantonio M, De Berardis D: **Relationship between Health-Anxiety and Cyberchondria: Role of Metacognitive Beliefs**. *J Clin Med* 2022, **11**(9).

44. Rashid Z, Rathore MA, Khushk IA, Mashhadi SF, Ahmed M, Shahzeb M: **Intolerance of Uncertainty and Anxiety Sensitivity as Prospective Risk Factors for Cyberchondria in Undergraduate Students**. *Annals of King Edward Medical University* 2022, **28**(1):91-96.

45. Santoro G, Starcevic V, Scalone A, Cavallo J, Musetti A, Schimmenti A: **The doctor is in (ternet): the mediating role of health anxiety in the relationship between somatic symptoms and cyberchondria**. *Journal of personalized medicine* 2022, **12**(9):1490.

46. Sezer Ö, Başoğlu MA, Dağdeviren HN: **An examination of cyberchondria’s relationship with trait anxiety and psychological well-being in women of reproductive age: A cross-sectional study**. *Medicine* 2022, **101**(46):e31503.

47. Vismara M, Benatti B, Ferrara L, Colombo A, Bosi M, Varinelli A, Pellegrini L, Viganò C, Fineberg NA, Dell’Osso B: **A preliminary investigation of cyberchondria and its correlates in a clinical sample of patients with obsessive–compulsive disorder, anxiety and depressive disorders attending a tertiary psychiatric clinic**. *International Journal of Psychiatry in Clinical Practice* 2022, **26**(2):111-122.

48. Yalçın İ, Boysan M, Eşkisu M, Çam Z: **Health anxiety model of cyberchondria, fears, obsessions, sleep quality, and negative affect during COVID-19**. *Curr Psychol* 2022:1-18.

49. Zhou Y, Dai L, Deng Y, Zeng H, Yang L: **The moderating effect of alexithymia on the relationship between stress and cyberchondria**. *Front Psychiatry* 2022, **13**:1043521.

50. Zolotareva A: **Cyberchondria, but not preventive behavior, mediates the relationship between fear of COVID-19 and somatic burden: Evidence from Russia**. *Front Psychiatry* 2022, **13**:1018659.

51. Błachnio A, Przepiórka A, Kot P, Cudo A, McElroy E: **The mediating role of rumination between stress appraisal and cyberchondria**. *Acta Psychologica* 2023, **238**:103946.

52. El-Zayat A, Namnkani SA, Alshareef NA, Mustfa MM, Eminaga NS, Algarni GA: **Cyberchondria and its Association with Smartphone Addiction and Electronic Health Literacy among a Saudi Population**. *Saudi J Med Med Sci* 2023, **11**(2):162-168.

53. Eşkisu M, Çam Z, Boysan M: **Health-Related Cognitions and Metacognitions Indirectly Contribute to the Relationships Between Impulsivity, Fear of COVID-19, and Cyberchondria**. *J Ration Emot Cogn Behav Ther* 2023:1-23.

54. Infanti A, Starcevic V, Schimmenti A, Khazaal Y, Karila L, Giardina A, Flayelle M, Razavi SBH, Baggio S, Vögele C: **Predictors of cyberchondria during the COVID-19 pandemic: cross-sectional study using supervised machine learning**. *JMIR formative research* 2023, **7**(1):e42206.

55. Jeong GC, Lee K, Jin Y: **Effects of the Fear of COVID-19 and Efficacy of Coping Behavior for Infectious Diseases after the End of COVID-19: Moderating Effects of Cyberchondria and eHealth Literacy**. *Behav Sci (Basel)* 2023, **13**(8).

56. Liu Y, Peng W, Cao M, Zhang S, Peng J, Zhou Z: **Cyberchondria and Chinese Adolescent Mental Health in the Age of COVID-19 Pandemic**. *Cyberpsychol Behav Soc Netw* 2023, **26**(8):631-639.

57. Nasiri M, Mohammadkhani S, Akbari M, Alilou MM: **The structural model of cyberchondria based on personality traits, health-related metacognition, cognitive bias, and emotion dysregulation**. *Frontiers in Psychiatry* 2023, **13**:960055.

58. Tarabay C, Bitar Z, Akel M, Hallit S, Obeid S, Soufia M: **Cyberchondria Severity and Quality of Life Among Lebanese Adults: The Moderating Effect of Emotions**. *Prim Care Companion CNS Disord* 2023, **25**(2).

59. Varer Akpinar C, Mandiracioglu A, Ozvurmaz S, Kurt F, Koc N: **Cyberchondria and COVID-19 anxiety and internet addiction among nursing students**. *Current Psychology* 2023, **42**(3):2406-2414.

60. Vujić A, Volarov M, Latas M, Demetrovics Z, Kiraly O, Szabo A: **Are Cyberchondria and Intolerance of Uncertainty Related to Smartphone Addiction?** *Int J Ment Health Addict* 2023:1-19.

61. Wang D, Sun L, Shao Y, Zhang X, Maguire P, Hu Y: **Research and Evaluation of a Cyberchondria Severity Scale in a Chinese Context**. *Psychol Res Behav Manag* 2023, **16**:4417-4429.

62. Yam FC, Korkmaz O, Griffiths MD: **The association between fear of Covid-19 and smartphone addiction among individuals: the mediating and moderating role of cyberchondria severity**. *Curr Psychol* 2023, **42**(3):2377-2390.

63. Zhu X, Zheng T, Ding L, Zhang X: **Exploring associations between eHealth literacy, cyberchondria, online health information seeking and sleep quality among university students: A cross-section study**. *Heliyon* 2023, **9**(6):e17521.

64. Agrawal V, Khulbe Y, Singh A, Kar SK: **The digital health dilemma: Exploring cyberchondria, well-being, and smartphone addiction in medical and non-medical undergraduates**. *Indian J Psychiatry* 2024, **66**(3):256-262.

65. Ali SS, Hendawi NE, El-Ashry AM, Mohammed MS: **The relationship between cyberchondria and health literacy among first-year nursing students: the mediating effect of health anxiety**. *BMC Nurs* 2024, **23**(1):776.

66. Atsizata M, Sögüt SC: **The relationship between orthorexia nervosa and cyberchondria levels in nurses: A cross-sectional study**. *Arch Psychiatr Nurs* 2024, **48**:30-35.

67. El-Zoghby SM, Zaghloul NM, Tawfik AM, Elsherbiny NM, Shehata SA, Soltan EM: **Cyberchondria and smartphone addiction: A correlation survey among undergraduate medical students in Egypt**. *J Egypt Public Health Assoc* 2024, **99**(1):7.

68. Fang J, Qiu C, Sun Z, Zhou J, He P, Conti A, Lu Y, Huang X, Xu J, Tang W: **A national survey of pandemic fear and cyberchondria after ending zero-COVID policy: The chain mediating role of alexithymia and psychological distress**. *Compr Psychiatry* 2024, **133**:152505.

69. Fang S, Mushtaque I: **The Moderating Role of Health Literacy and Health Promoting Behavior in the Relationship Among Health Anxiety, Emotional Regulation, and Cyberchondria**. *Psychology Research and Behavior Management* 2024:51-62.

70. Jungmann SM, Gropalis M, Schenkel SK, Witthöft M: **Is cyberchondria specific to hypochondriasis?** *Journal of Anxiety Disorders* 2024, **102**:102798.

71. Kalantari A, Valizadeh-Haghi S, Starcevic V, Shahbodaghi A, Rahmatizadeh S, Zayeri F, Khazaal Y: **The relationship between e-Health literacy and cyberchondria in Iranian students of health sciences**. *Front Psychiatry* 2024, **15**:1421391.

72. Mrayyan MT, Abu Khait A, Al-Mrayat Y, Alkhawaldeh JfM, Alfayoumi I, Algunmeeyn A, Kutah OA, Abunab HY, Hamdan MS, Alhabashneh H: **Anxiety sensitivity moderates the relationship between internet addiction and cyberchondria among nurses**. *Journal of Health Psychology* 2024:13591053241249634.

73. Sansakorn P, Mushtaque I, Awais EYM, Dost MKB: **The Relationship between Cyberchondria and Health Anxiety and the Moderating Role of Health Literacy among the Pakistani Public**. *Int J Environ Res Public Health* 2024, **21**(9).

74. Šoštarić M, Jokić-Begić N, Vukušić Mijačika M: **Can't stop, won't stop - understanding anxiety's role in cyberchondria among pregnant women**. *Women Health* 2024, **64**(2):185-194.

75. Xu RH, Chen C: **Moderating Effect of Coping Strategies on the Association Between the Infodemic-Driven Overuse of Health Care Services and Cyberchondria and Anxiety: Partial Least Squares Structural Equation Modeling Study**. *Journal of Medical Internet Research* 2024, **26**:e53417.

76. Amanak K, Şule Bilgiç F: **Cyberchondria and pregnancy-related anxiety: multidimensional assessment of Health anxiety, sensitivity, uncertainty, and fear of childbirth in pregnant women**. *Psychol Health Med* 2025:1-15.

77. Cao Z, Sun Y, Li H, Lin C, Wong HL, Ming H, Wo PK, Wang J, Xiong X: **From Cyberchondria to Temporomandibular Disorders: How Somatic Symptoms and Anxiety Bridge the Gap**. *J Oral Rehabil* 2025, **52**(10):1777-1787.

78. Cіcі R, Topdemіr EA, Kapikiran G: **The Relationship Between Preoperative Cyberchondria Levels and Death Anxiety of Liver Donors**. *Nurs Health Sci* 2025, **27**(3):e70179.

79. Demir G, Bahar Z, Yildirim D: **Evaluation of health anxiety and cyberchondria levels in adolescent high school students**. *J Child Adolesc Ment Health* 2025:1-17.

80. Ergün Özdel ZG, Özkaya G, Türe Ş: **Parental health information seeking online: How cyberchondria and health literacy shape pediatric health decisions**. *Arch Argent Pediatr* 2025:e202510771.

81. Kefeli Col B, Gumusler Basaran A, Genc Kose B: **The Relationship Between E-Health Literacy, Health Anxiety, Cyberchondria, and Death Anxiety in University Students That Study in Health Related Department**. *J Multidiscip Healthc* 2025, **18**:1581-1595.

82. Lai YK, Lai Z, Zhao X: **Counteracting cyberchondria in Chinese chronic disease patients: The divergent roles of health-related social media use and online patient-centered communication**. *Patient Educ Couns* 2025, **141**:109337.

83. Macovei M, Măirean C: **Health anxiety and death anxiety: The role of cyberchondria and social aspirations**. *J Health Psychol* 2025:13591053251341191.

84. Xu RH, Liang X, Starcevic V: **Exploring the Relationship Between Cyberchondria and Suicidal Ideation: Cross-Sectional Mediation Analysis**. *J Med Internet Res* 2025, **27**:e72414.

85. Xu RH, Starcevic V: **Cyberchondria in Older Adults and Its Relationship With Cognitive Fusion, Health-Related Quality of Life, and Mental Well-Being: Mediation Analysis**. *JMIR Aging* 2025, **8**:e70302.

86. Yorulmaz M, Göde A, Aydoğdu A, Dilekçi R: **Investigation of the effect of internet addiction on cyberchondria**. *Psychol Health Med* 2025, **30**(6):1187-1198.

87. Yurttaş M, İzgi E, Gürbüz E: **Analysis of relationship between cyberchondria levels and oral health in dental patients**. *BMC Oral Health* 2025, **25**(1):1476.
